# Mitochondrial DNA hyperdiversity and its potential causes in the marine periwinkle *Melarhaphe neritoides* (Mollusca: Gastropoda)

**DOI:** 10.7717/peerj.2549

**Published:** 2016-10-05

**Authors:** Séverine Fourdrilis, Patrick Mardulyn, Olivier J. Hardy, Kurt Jordaens, António Manuel de Frias Martins, Thierry Backeljau

**Affiliations:** 1 Directorate Taxonomy and Phylogeny & JEMU, Royal Belgian Institute of Natural Sciences, Brussels, Belgium; 2 Evolutionary Biology and Ecology, Université Libre de Bruxelles, Brussels, Belgium; 3 Department of Biology, Invertebrate Section, Royal Museum for Central Africa, Tervuren, Belgium; 4 CIBIO, Centro de Investigação em Biodiversidade e Recursos Genéticos, InBIO Laboratório Associado, Pólo dos Açores, Departamento de Biologia da Universidade dos Açores, University of the Azores, Ponta Delgada, Portugal; 5 Evolutionary Ecology Group, University of Antwerp, Antwerp, Belgium

**Keywords:** mtDNA hyperdiversity, Haplotype diversity, Nucleotide diversity, Planktonic dispersal, Effective population size, Selection, Mutation rate

## Abstract

We report the presence of mitochondrial DNA (mtDNA) hyperdiversity in the marine periwinkle *Melarhaphe neritoides* (Linnaeus, 1758), the first such case among marine gastropods. Our dataset consisted of concatenated 16S-COI-Cyt*b* gene fragments. We used Bayesian analyses to investigate three putative causes underlying genetic variation, and estimated the mtDNA mutation rate, possible signatures of selection and the effective population size of the species in the Azores archipelago. The mtDNA hyperdiversity in *M. neritoides* is characterized by extremely high haplotype diversity (*Hd* = 0.999 ± 0.001), high nucleotide diversity (*π* = 0.013 ± 0.001), and neutral nucleotide diversity above the threshold of 5% (*π*_syn_ = 0.0677). Haplotype richness is very high even at spatial scales as small as 100*m*^2^. Yet, mtDNA hyperdiversity does not affect the ability of DNA barcoding to identify *M. neritoides*. The mtDNA hyperdiversity in *M. neritoides* is best explained by the remarkably high mutation rate at the COI locus (*μ* = 5.82 × 10^−5^ per site per year **or *μ* = 1.99 × 10^−4^ mutations per nucleotide site per generation), whereas the effective population size of this planktonic-dispersing species is surprisingly small (*N*_*e*_ = 5, 256; CI = 1,312–3,7495) probably due to the putative influence of selection. Comparison with COI nucleotide diversity values in other organisms suggests that mtDNA hyperdiversity may be more frequently linked to high *μ* values and that mtDNA hyperdiversity may be more common across other phyla than currently appreciated.

## Introduction

The term DNA hyperdiversity is usually applied to populations when neutral nucleotide diversity at selectively unconstrained synonymous sites is ≥5% (Cutter, Jovelin & Dey, 2013), that is when two 100 bp protein-coding DNA sequences (mitochondrial or nuclear) chosen randomly from a population sample differ on average at five or more synonymous and neutral nucleotide positions. Nucleotide diversity in a sequence alignment is calculated either from pairwise differences at all sites (*π*) or at segregating sites only (*θ*) (Nei, 1987; Nei & Miller, 1990; Watterson, 1975). Yet, *π* is often preferred because its estimation is less sensitive to sequencing errors and DNA sequence length than *θ* (Johnson & Slatkin, 2008). Nucleotide diversity is also calculated at synonymous sites (*π*_syn_) to obtain an estimate of neutral polymorphism reflecting the balance between mutation pressure and genetic drift. This latter measure of *π*_syn_ is required to observe hyperdiversity. DNA hyperdiversity is usually associated with fast evolving prokaryotes and viruses and less frequently with eukaryotic organisms showing lower rates of evolution (Drake et al., 1998). Nevertheless, mitochondrial (mtDNA) or nuclear (nDNA) DNA data retrieved from literature references on 505 animal species, showed signatures of DNA hyperdiversity (*π*_syn_ ≥ 0.05) in 43% of the species studied, i.e., 42% among 394 Chordata, 55% among 66 Arthropoda, 33% among 24 Mollusca, 24% among 17 Echinodermata, and 100% among 3 Nematoda (Table S1). Although these percentages most probably reflect strong sampling bias, DNA hyperdiversity seems not uncommon in eukaryotes. Rates of mtDNA evolution are 10–30 times faster than nDNA and drive mitonuclear coevolution and speciation through strong selection pressure (Blier, Dufresne & Burton, 2001; Hill, 2016; Lane, 2009). Hyperdiverse intraspecific mtDNA variation provides a greater density of polymorphic sites for selection to act upon (Cutter, Jovelin & Dey, 2013), and possibly provokes higher speciation rate as observed in birds and reptiles (Eo & DeWoody, 2010). Studying mtDNA hyperdiversity is hence interesting to better understand how evolutionary processes such as mutational dynamics and selection that underlie mitonuclear coevolution contribute to speciation (Burton & Barreto, 2012).

mtDNA is a popular population genetic marker because of its variability and, as such, is widely used for evolutionary studies at the species level (Féral, 2002; Wan et al., 2004) and DNA barcoding (Hebert, Ratnasingham & DeWaard, 2003). The main determinants of animal mtDNA diversity are supposed to be mutation rate (*μ*) and selection, while in contrast to nDNA, effective population size (*N*_*e*_) and ecology (life history traits) are expected to be less important (Bazin, Glémin & Galtier, 2006; Cutter, Baird & Charlesworth, 2006; Dey et al., 2013; Lanfear, Kokko & Eyre-Walker, 2014; Leffler et al., 2012; Nabholz, Glémin & Galtier, 2009; Nabholz et al., 2008; Small et al., 2007). The higher nDNA diversity observed in invertebrates vs. vertebrates, in marine vs. non-marine species, and in small vs. large organisms, is probably therefore not in line with patterns of mtDNA diversity (Bazin, Glémin & Galtier, 2006; Leffler et al., 2012). As the main determinants of animal mtDNA diversity are supposed to be *μ* and selection, mtDNA hyperdiversity is more likely to be also explained by high *μ* or selection on the mitochondrial genome, and we expect that the relationship between *N*_*e*_ and mtDNA diversity may be weakened. Still, at least in eutherian mammals and reptiles mtDNA diversity seems to correlate with *N*_*e*_ (Hague & Routman, 2016; Mulligan, Kitchen & Miyamoto, 2006), so that an eventual influence of *N*_*e*_ on mtDNA hyperdiversity cannot a priori be neglected. Hence, in summary, mtDNA diversity may be affected by amongst others: (1) mutations generating new alleles that increase mtDNA diversity, (2) diversifying and balancing selection that increase mtDNA diversity by favoring extreme or rare phenotypes (Maruyama & Nei, 1981; Mather, 1955; Rueffler et al., 2006), (3) other types of selection that decrease mtDNA diversity by eliminating disadvantageous alleles (Anisimova & Liberles, 2012), and (4) fluctuations in *N*_*e*_ since more mutations arise in populations with larger *N*_*e*_ (Kimura, 1983). Yet, far more empirical data are needed to better understand the relative contribution of various determinants of mtDNA hyperdiversity.

In the present work, we investigate three potential determinants of mtDNA hyperdiversity i.e., *μ*, selection and *N*_*e*_, in the marine periwinkle *Melarhaphe neritoides* (Linnaeus, 1758) in the Azores archipelago. *Melarhaphe neritoides* is an intertidal gastropod that shows signatures of mtDNA hyperdiversity (see data in García et al., 2013). It is a small (shell up to 11 mm) temperate species (Lysaght, 1941), in which the sedentary adults produce pelagic egg capsules and long-lived planktonic larvae with high dispersal potential during 4–8 weeks until settlement (Cronin, Myers & O’riordan, 2000; Fretter & Manly, 1977; Lebour, 1935). *Melarhaphe neritoides* is widely distributed throughout Europe (Fretter & Graham, 1980), where it shows a remarkable macrogeographic population genetic homogeneity (inferred from allozyme data) (Johannesson, 1992), though locally in Spain it displays huge amounts of mtDNA COI diversity in terms of a large numbers of polymorphic sites (*S* = 16%), a very high haplotype diversity (*Hd* = 0.998) and a very high nucleotide diversity (*π* = 0.019) (García et al., 2013). We studied mtDNA diversity of *M. neritoides* within the archipelago of the Azores because this area provides a vast, though relatively isolated, setting to explore geographic mtDNA variation at different spatial scales.

First, we formally describe and evaluate mtDNA hyperdiversity in *M. neritoides*, by assessing diversity in three mtDNA gene fragments, *viz.* 16S ribosomal RNA (16S), cytochrome oxidase c subunit I (COI) and cytochrome b (Cyt*b*) in substantial numbers of individuals and locations. Second, we survey the literature to compare *M. neritoides* mtDNA hyperdiversity with other littorinids, other planktonic-dispersing gastropods showing high genetic diversity, and other hyperdiverse molluscs in general. Finally, we explore the relationship between mtDNA diversity in *M. neritoides* and (1) *μ*, (2) selection, (3) *N*_*e*_, (4) population genetic structuring, and (5) phylogeny.

## Materials and Methods

### Samples and DNA collection

**Figure 1:**
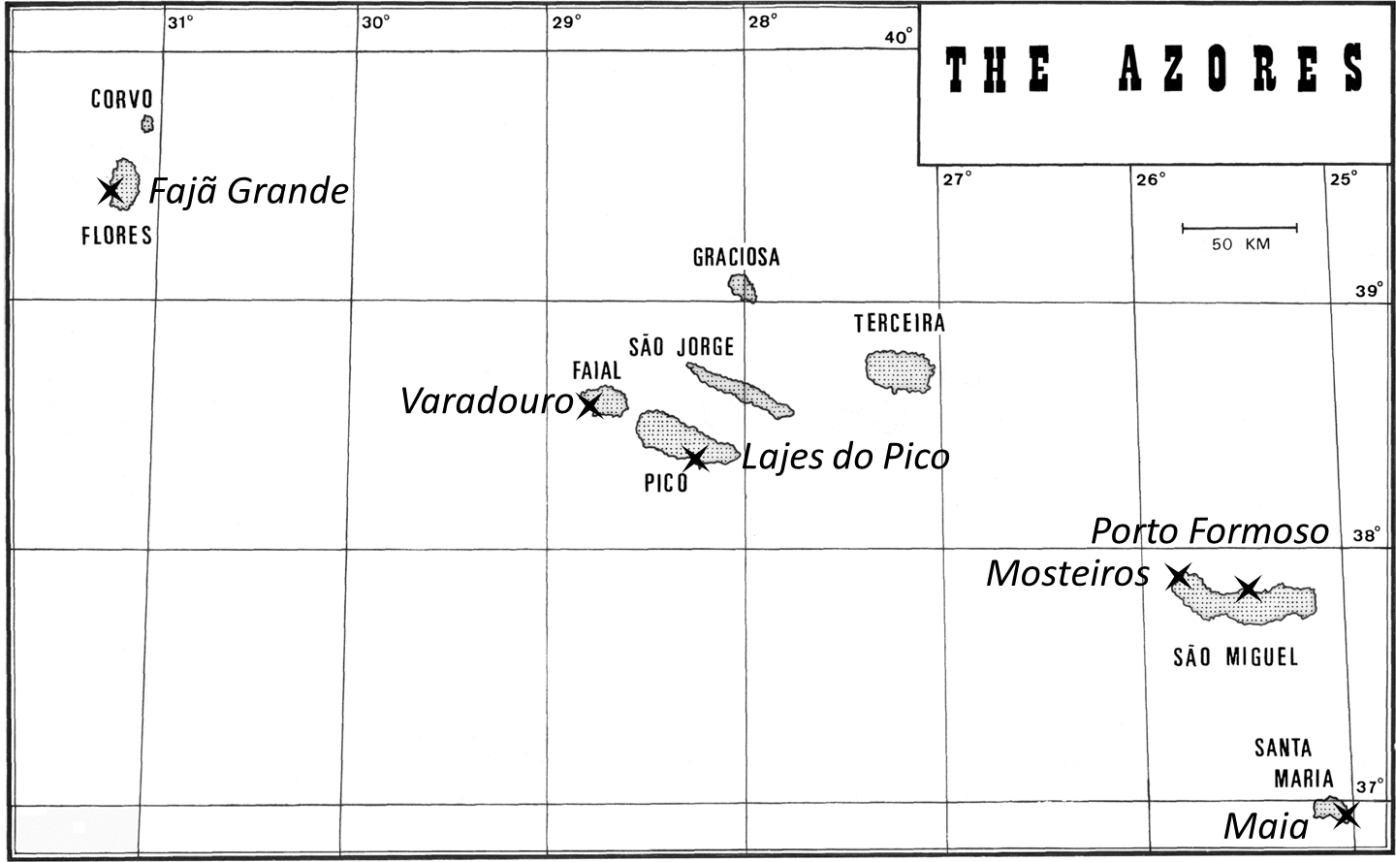
Sampling sites (cross-shaped symbols) of *M. neritoides* in the Azores archipelago, Portugal. FAI, Varadouro, Faial island; FLO, Fajã Grande, Flores island; MOS, Mosteiros, São Miguel island; PIC, Lajes do Pico, Pico island; SMA, Maia, Santa Maria island; SMI, Porto Formoso, São Miguel island.

A total of 610 specimens of *M. neritoides* were collected between 1992 and 2012 at six localities in the Azores archipelago, Portugal, *viz.* Varadouro, Faial island (FAI), Fajã Grande, Flores island (FLO), Mosteiros, São Miguel island (MOS), Lajes do Pico, Pico island (PIC), Maia, Santa Maria island (SMA), and Porto Formoso, São Miguel island (SMI) (Fig. 1). These 610 specimens contribute to our analyzed data sets as follows (Table S2): (1) dataset 1: 185 specimens from five islands sequenced for COI (614 bp), 16S (482 bp) and Cyt*b* (675 bp) to investigate mtDNA diversity and demographic history; (2) dataset 2: 223 specimens from one island collected at a single spot of about 100 m^2^ at MOS and sequenced for COI (657 bp) and 213 among these sequenced for 16S (482 bp), to assess microscale mtDNA haplotype richness; (3) dataset 3: 169 specimens from four islands collected between 1992 and 1993, and 175 specimens collected in 2012 at the same four localities, sequenced for COI (578 bp) to generate a temporal series of samples over 20 years for estimating mtDNA *μ*; (4) dataset 4: 212 specimens from five islands sequenced for COI (605 bp), completed by one COI sequence of *M. neritoides* from the United Kingdom retrieved from GenBank (AJ488608) and 86 COI sequences of seven species from the three littorinid subfamilies Lacuninae, Laevilitorininae and Littorininae (Reid, Dyal & Williams, 2012) and one species of Pomatiidae available in GenBank, viz. *Bembicium auratum* (Lacuninae) (AJ488606), *Cremnoconchus syhadrensis* (Lacuninae) (AJ488605), *Lacuna pallidula* (Lacuninae) (AJ488604, KT996151), *Laevilitorina caliginosa* (Laevilitorininae) (AJ488607), *Littorina littorea* (Littorininae) (AJ622946, HM884235, HM884236, HM884248, KF643337, KF643416, KF643449, KF643454, KF643456, KF643464, KF643631, KF643658, KF643697, KF643729, KF643906, KF644042, KF644180, KF644262, KF644330), *Peasiella isseli* (Littorininae) (HE590849), *Pomatias elegans* (Pomatiidae) (JX911283, JQ964789, GQ424199, EU239237, EU239238, EU239239, EU239240, EU239241) and *Tectarius striatus* (Littorininae) (DQ022012 –DQ022064), to assess monophyly, possible phylogenetic structuring, and eventual cryptic taxonomic diversity in *M. neritoides*.

Collected specimens were preserved at −20 °C until DNA analysis. Individual genomic DNA was extracted from foot muscle following the standard protocol of either the NucleoSpin^®^ Tissue kit (Macherey-Nagel GmbH & Co. KG, Düren, Germany) or the DNeasy 96 Blood & Tissue kit (Qiagen GmbH, Hilden, Germany). Remaining soft body parts and shells have been deposited in the collections of the Royal Belgian Institute of Natural Sciences, Brussels (RBINS) under the general inventory number IG 32962.

### 16S, COI and Cyt*b* amplification and sequence alignment

PCR amplification was carried out in a 20-µL reaction volume using standard Taq DNA polymerase (Qiagen GmbH, Hilden, Germany) and universal primers LCO1490 (5′-GGTCAACAAATCATAAAGATATTGG-3′) and HCO2198 (5′-TAAACTTCAGGGT GACCAAAAAATCA-3′) for a 578-to-657 bp region of COI (Folmer et al., 1994), universal primers 16Sar (5′-CGCCTGTTTAACAAAAACAT-3′) and 16Sbr (5′-CCGGTCTGAA CTCAGATCACGT-3′) for a 482 bp region of 16S (Simon et al., 1994), and littorinid-specific primers 14825 (5′-CCTTCCCGCACCTTCAAATC-3′) and 15554 (5′-GCAAATAAAAAG TATCACTCTGG-3′) for a 675 bp region of Cyt*b* (Reid, Rumbak & Thomas, 1996). The PCR conditions for COI consisted of an initial denaturation at 95 °C for 5 min, 40 cycles of denaturation at 95 °C for 45 s, annealing at 45 °C for 45 s, elongation at 72 °C for 1 min 30 s, and a final elongation at 72 °C for 10 min. The PCR conditions for Cyt*b* were the same except for the annealing step at 48 °C. The PCR conditions for 16S were also the same except for the annealing step at 52 °C, 35 cycles instead of 40, and final elongation for 5 min. PCR products were purified using Exonuclease I and FastAP Thermosensitive Alkaline Phosphatase (Thermo Scientific, Erembodegem-Aalst, Belgium). Sequencing reactions were performed directly on purified PCR products using the BigDye^®^ Terminator v1.1 Cycle Sequencing kit (Life Technologies, Gent, Belgium) and run on an Applied Biosystems 3130xl Genetic Analyser automated capillary sequencer, or outsourced to Macrogen (Rockville, MD, USA). Sample files were assembled, edited and reviewed using ABI Prism^®^ SeqScape^®^ 2.5.0 (Applied Biosystems). The accuracy and reproducibility of the PCR results were validated by triplicating COI and 16S amplifications on a subset of 20 individuals, using standard Taq DNA polymerase for two replicates and HotStar HiFidelity DNA Polymerase (Qiagen GmbH, Hilden, Germany) for one replicate. Sequence alignments were made with ClustalW (Thompson, Higgins & Gibson, 1994) using default parameters in BioEdit 7.0.9.0 (Hall, 1999). All sequences were deposited in GenBank (KT996151 –KT997344). The morphology-based identification of *M. neritoides* was validated through DNA barcoding by querying the 185 COI fragments from dataset 1 in the Barcode of Life Data systems (BOLD) (Ratnasingham & Hebert, 2007).

### mtDNA diversity

The three gene fragments were concatenated for the 185 specimens of dataset 1, using Geneious 5.3.4 (http://www.geneious.com, Kearse et al., 2012). DNA diversity metrics (Tables 1 and 2) were calculated with DnaSP 5.10.1 (Librado & Rozas, 2009). Despite the fact that 32–42 specimens were sampled per site, there were no shared haplotypes between sampling sites (*H*_*s*_ = 0), i.e., all haplotypes were private (Table 1). Consequently, we examined mtDNA haplotype richness at a microscale, i.e., within a sampling site. Dataset 2, composed of identical fragment lengths across individuals, was used to compute individual-based rarefaction curves for the COI and 16S fragments using EstimateS 8.2.0 (Colwell, 2006) in order to assess the relationship between the number of haplotypes observed (*H*_obs_) and sample size, and compute the *Chao1* and *Chao2* richness estimators (Chao, 1984; Chao, 1987). Given that COI and Cyt*b* showed similar diversity levels (Table 1), only COI was used for rarefaction analysis. A logarithmic trendline, best fitting the data, was applied to each rarefaction curve to extrapolate *H*_obs_ for larger sample sizes.

**Table 1:** mtDNA diversity metrics of *Melarhaphe neritoides*. Statistics describing the number of individuals (*N*), number of haplotypes (*H*), number of private haplotypes (*H*_*p*_), number of shared haplotypes among sampling sites (*H*_*s*_), number of shared haplotypes within sampling site (*H*_*w*_), DNA fragment length in base pairs (*L*), number of segregating sites (*S*) and its corresponding percentage of the fragment length into brackets, haplotype diversity (*Hd*) ± standard deviation, Jukes-Cantor corrected nucleotide diversity (*π*) ± standard deviation, Jukes-Cantor corrected nucleotide diversity at synonymous sites (*π*_syn_) and Jukes-Cantor corrected nucleotide diversity at non-synonymous sites (*π*_non-syn_).

	*N*	*H*	*H*_*p*_	*H*_*s*_	*H*_*w*_	*L*	*S*	*Hd*	*π*	*π*_syn_	*π*_non-syn_
16S-COI-Cyt*b*	185	184	184	0	1	1,771	420 (24%)	0.999 ± 0.001	0.013 ± 0.001	0.0677	0.0004
16S	185	77	63	12	2	482	71 (15%)	0.814 ± 0.030	0.004 ± 0.001	–	–
COI	185	156	142	13	1	614	169 (28%)	0.996 ± 0.002	0.018 ± 0.001	0.0736	0.0001
Cyt*b*	185	166	153	9	4	675	180 (27%)	0.998 ± 0.001	0.016 ± 0.001	0.0637	0.0006

**Table 2:** Overview of mtDNA diversity in other Littorinidae, various highly diverse planktonic-dispersers and hyperdiverse mollusc species. Taxa are listed by decreasing value of haplotype diversity.

Species		Larval development	Sampling area	*N*	Locus	*L*	*Hd*	*π*	Reference
**Other Littorinidae**									
**Temperate species**									
*Littorina saxatilis*	Mo	d	North Atlantic	453	ND1-tRNApro-ND6-Cyt*b*	1,154	0.940	0.005	Doellman et al. (2011)
			North Atlantic	778	Cyt*b*	607	0.905	0.009	Panova et al. (2011)
*Tectarius striatus*	Mo	p (unknown)	Macaronesia	109	COI-Cyt*b*	993	0.934	0.006	Van den Broeck et al. (2008)
*Littorina keenae*	Mo	p (unknown)	North Pacific	584	ND6-Cyt*b*	762	0.815	0.003	Lee & Boulding (2007)
*Littorina littorea*	Mo	p (28–42 days)	North Atlantic	488	COI	424	0.810	0.004	Calculated from data in Wares et al. (2002), Williams, Reid & Littlewood (2003), Williams & Reid (2004), Giribet et al. (2006), Blakeslee, Byers & Lesser (2008), Layton, Martel & Hebert (2014)
*Littorina plena*	Mo	p (64 days)	NE Pacific	135	Cyt*b*	414	0.775	0.006	Lee & Boulding (2009)
*Littorina obtusata*	Mo	d	North Atlantic	46	COI	582	0.762	0.006	Calculated from data in Wares & Cunningham (2001)
			NW Atlantic	31	COI	574	0.127	0.001	Calculated from data in Layton, Martel & Hebert (2014)
*Bembicium vittatum*	Mo	d	Indian Ocean	40	12S	324	0.730	–	Kennington, Hevroy & Johnson (2012)
*Austrolittorina unifasciata*	Mo	p (4 weeks)	Australia	102	COI	658	0.541	0.002	Calculated from data in Colgan et al. (2003), Williams, Reid & Littlewood (2003), Waters, Mcculloch & Eason (2007)
*Littorina scutulata*	Mo	p (37–70 days)	NE Pacific	265	Cyt*b*	414	0.389	0.003	Lee & Boulding (2009)
*Littorina subrotundata*	Mo	d	NE Pacific	229	Cyt*b*	414	0.297	0.001	Lee & Boulding (2009)
*Austrolittorina antipodum*	Mo	p (4 weeks)	New Zealand	40	COI	658	0.146	0.001	Calculated from data in Williams, Reid & Littlewood (2003), Waters, Mcculloch & Eason (2007)
*Littorina sitkana*	Mo	d	NE Pacific	146	Cyt*b*	414	0.093	0.001	Lee & Boulding (2009)
**Tropical species**									
*Echinolittorina reticulata*	Mo	p (3–4 weeks)	Indo-Pacific	37	COI	1,251	1.000	0.009	Reid et al. (2006)
*Echinolittorina vidua*	Mo	p (3–4 weeks)	Indo-Pacific	92	COI	1,217	0.996	0.041	Reid et al. (2006)
*Echinolittorina trochoides C*	Mo	p (3–4 weeks)	Indo-Pacific	14	COI	1,251	0.989	0.006	Reid et al. (2006)
*Littoraria coccinea glabrata*	Mo	p (unknown)	Indian Ocean	45	COI	451	0.954	0.006	Silva et al. (2013)
*Echinolittorina trochoides A*	Mo	p (3–4 weeks)	Indo-Pacific	46	COI	1,251	0.943	0.009	Reid et al. (2006)
*Echinolittorina trochoides B*	Mo	p (3–4 weeks)	Indo-Pacific	18	COI	1,251	0.935	0.004	Reid et al. (2006)
*Bembicium nanum*	Mo	p (weeks)	Australia	54	COI	806	0.920	0.006	Ayre, Minchinton & Perrin (2009)
*Echinolittorina trochoides E*	Mo	p (3–4 weeks)	Indo-Pacific	21	COI	1,251	0.900	0.003	Reid et al. (2006)
*Echinolittorina trochoides D*	Mo	p (3–4 weeks)	Indo-Pacific	20	COI	1,251	0.884	0.003	Reid et al. (2006)
*Cenchritis muricatus*	Mo	p (4 weeks)	Caribbean	77	COI	282	0.850	0.008	Díaz-Ferguson et al. (2012)
*Echinolittorina ziczac*	Mo	p (3–4 weeks)	Caribbean Sea	31	COI	431	0.750	0.004	Díaz-Ferguson et al. (2012)
*Echinolittorina lineolata*	Mo	p (3–4 weeks)	South Atlantic	496	COI	441	0.704	0.003	Calculated from Genbank data KJ857561 –KJ858054 and Williams & Reid (2004)
			South Atlantic	442	Cyt*b*	203	0.284	0.002	Calculated from Genbank data KM210838 –KM211279
*Littoraria scabra*	Mo	p (unknown)	Indo- Pacific	50	COI	527	0.690	0.003	Silva et al. (2013)
*Littoraria irrorata*	Mo	p (4 weeks)	NE Atlantic	238	COI	682	0.546	0.004	Calculated from data in Díaz-Ferguson et al. (2010), Robinson et al. (2010), Reid, Dyal & Williams (2010)
**Other highly diverse planktonic-dispersing marine invertebrates**									
*Glaucus atlanticus*	Mo	p (lifelong)	Worldwide	112	COI	658	0.996	0.014	calculated from data in Churchill, Alejandrino & Valdés (2013), Churchill, Valdés & Ó Foighil (2014), Wecker et al. (2015)
*Pygospio elegans*	An	p (4–5 weeks)	North Sea	23	COI	600	0.996	0.014	Kesäniemi, Geuverink & Knott (2012)
*Argopecten irradians concentricus*	Mo	p (5–19 days)	NW Atlantic	219	mtDNA	1,025	0.982	0.008	Marko & Barr (2007)
*Brachidontes pharaonis*	Mo	p (weeks)	Mediterranean-Red Sea	34	COI	618	0.973	0.039	Terranova et al. (2007)
*Ruditapes philippinarum*	Mo	p (2–3 weeks)	NW Pacific	170	COI	644	0.960	0.010	Mao et al. (2011)
*Cellana sandwicensis*	Mo	p (4 days)	Hawaii	109	COI	612	0.960	0.006	Bird et al. (2007)
*Holothuria nobilis*	Ec	p (13–26 days)	Indo-Pacific	360	COI	559	0.942	0.008	Uthicke & Benzie (2003)
*Tridacna maxima*	Mo	p (9 days)	Indo-Pacific	211	COI	484	0.940	0.023	Nuryanto & Kochzius (2009))
*Tridacna crocea*	Mo	p (1 week)	Indo-Malaysia	300	COI	456	0.930	0.015	Kochzius & Nuryanto (2008))
*Pachygrapsus crassipes*	Ar	p (95 days)	NE Pacific	346	COI	710	0.923	0.009	Cassone & Boulding (2006)
*Tripneustes gratilla*	Ec	p (18 days)	Indo-Pacific	83	COI	573	0.902	0.004	calculated from data in Lessios, Kane & Robertson (2003)
*Holothuria polii*	Ec	p (13–26 days)	Mediterranean Sea	158	COI	484	0.873	0.005	Vergara-Chen et al. (2010)
*Nacella magellanica*	Mo	p (unknown)	SW Atlantic	171	COI	573–650	0.868	0.004	Aranzamendi, Bastida & Gardenal (2011)
*Bursa fijiensis*	Mo	p (8 weeks)	SW Pacific	59	COI	566	0.848	0.003	Castelin et al. (2012)
*Acropora cervicornis*	Cn	p (4 days)	Caribbean	160	mtCR	941	0.847	0.006	Vollmer & Palumbi (2007)
**Other hyperdiverse mollusc species**									
*Pliocardia kuroshimana*	Mo	p	NW Pacific	3	mtDNA	513	1.000	0.256^*^	James, Piganeau & Eyre-Walker (2016)
*Bulinus forskalii*	Mo	–	–	12	mtDNA	339	1.000	0.167^*^	James, Piganeau & Eyre-Walker (2016)
*Pyrgulopsis intermedia*	Mo	d	–	15	mtDNA	528	0.924	0.148^*^	James, Piganeau & Eyre-Walker (2016)
*Euhadra brandtii*	Mo	n/a	–	14	mtDNA	558	0.989	0.098^*^	James, Piganeau & Eyre-Walker (2016)
*Biomphalaria glabrata*	Mo	d	–	7	mtDNA	579	0.714	0.092^*^	James, Piganeau & Eyre-Walker (2016)
*Achatinella mustelina*	Mo	n/a	–	69	mtDNA	675	0.992	0.078^*^	James, Piganeau & Eyre-Walker (2016)
*Quincuncina infucata*	Mo	d	–	5	mtDNA	453	1.000	0.067^*^	James, Piganeau & Eyre-Walker (2016)
*Pyrgulopsis thompsoni*	Mo	d	–	7	mtDNA	657	0.952	0.066^*^	James, Piganeau & Eyre-Walker (2016)

**Notes.**

An Annelida Ar Arthropoda Cn Cnidaria; Ec Echinodermata Mo Mollusca d direct larval development p planktonic larval development (pelagic larval duration given in parenthesis) n/a not applicable *N* number of individuals *L* locus length in base pairs *Hd* haplotype diversity *π* nucleotide diversity

– missing data.

* *π* calculated at synonymous sites only (*π*_syn_).

### Population genetic structure

The monophyly of *M. neritoides* was assessed, and p-distances were compared within and among clades, in order to detect possible cryptic taxa and/or phylogenetic structuring that might contribute to the overall mtDNA hyperdiversity. First, two species trees were produced from dataset 4 using Bayesian inference (BI) and Maximum Likelihood (ML). Seven Littorinidae species were added to the ingroup. The outgroup *Pomatias elegans* belongs to a different family (Pomatiidae), but the same superfamily (Littorinoidea) as *M. neritoides*. Two independent runs of BI were performed using MrBayes 3.2.2 (Ronquist et al., 2012) hosted on the CIPRES Science Gateway (Miller, Pfeiffer & Schwartz, 2010), under a GTR + G nucleotide substitution model selected according to jModelTest 2.1.4 (Darriba et al., 2012), for 4.10^6^ generations with a sample frequency of 100 and a 30% burn-in. Convergence between the two runs onto the stationary distribution was assessed by examining whether the potential scale-reduction factors was close to 1 in the pstat file, standard deviation of split frequencies fell below 0.01 in the log file, and trace plots showed no trend by examining the p files in TRACER 1.6 (Rambaut et al., 2014). The final consensus tree was computed from the combination of both runs. ML analysis based on the GTR+G model was conducted in MEGA 6.06 (Tamura et al., 2013), with bootstrap consensus trees inferred from 1,000 replicates. Second, three methods of species delimitation were used: (1) the Automatic Barcode Gap Discovery (ABGD, available at http://wwwabi.snv.jussieu.fr/public/abgd/abgdweb.html) method (Puillandre et al., 2012), (2) the Bayesian implementation of the Poisson tree Processes (bPTP, available at http://species.h-its.org/ptp/) model (Zhang et al., 2013), and (3) the General Mixed Yule Coalescent (GMYC, available at http://species.h-its.org/gmyc/) model (Fujisawa & Barraclough, 2013; Pons et al., 2006). Finally, sequence divergence within and between clades was assessed by calculating mean within-group p-distances for the four species comprising more than one sequence (*Littorina littorea*, *N* = 19; *M. neritoides*, *N* = 213; *Pomatias elegans*, *N* = 8; *Tectarius striatus*, *N* = 53), and mean between groups p-distances for all species pairs (Table S2), using MEGA. Additionally, COI sequence divergence within *M. neritoides* was assessed by generating an intraspecific p-distances distribution from the 185 COI sequences included in dataset 1, using MEGA.

Dataset 1 was subjected to the program ALTER (http://sing.ei.uvigo.es/ALTER/, Glez-Peña et al., 2010) to convert the Fasta-formatted sequence alignment to a sequential Nexus-formatted file, which then could be analyzed by NETWORK 4.6.1.2 (www.fluxus-engineering.com, Bandelt, Forster & Röhl, 1999) to reconstruct a median-joining haplotype network. Population genetic structure in *M. neritoides* was qualitatively investigated with the haplotype network which provides information about phylogeographic structure and gene flow among populations, and quantified by *G*_ST_ (Pons & Petit, 1995), *N*_ST_ based on a distance matrix of pairwise differences (Pons & Petit, 1996) and φ_ST_ (Excoffier, Smouse & Quattro, 1992) using dataset 1 in SPAGEDI 1.4 (Hardy & Vekemans, 2002) for *G*_ST_ and *N*_ST_ and ARLEQUIN 3.5.1.3 (Excoffier & Lischer, 2010) for φ_ST_.

### mtDNA mutation rate

mtDNA evolves fast enough to provide sufficient variation for the estimation of *μ* over a two-decades period (Drummond et al., 2003), i.e., the time span of our temporal sampling and corresponding to 5–6 generations of *M. neritoides*. Dataset 3 comprises different sampling points in time, allowing sequences to be treated as heterochronous data for estimating the number of mutations occurring in the time interval between samples as described in Seo et al. (2002) and Drummond et al. (2002). In this way, the mutation rate per nucleotide site per year can be inferred using a Bayesian MCMC method as implemented in BEAST 2.1.3 (Bouckaert et al., 2014) hosted on the CIPRES Science Gateway. The Bayesian MCMC analysis was performed under a HKY substitution model (the closest model to GTR since GTR is not available in BEAST) with empirical base frequencies and a fixed substitution rate of 1.0 and a tree prior set to “*coalescent exponential population*” (chosen after model comparison with the “*coalescent constant population*” and the “*coalescent Bayesian skyline*” priors), a strict clock model assuming a constant substitution rate over time and a prior set to lognormal with *M* = − 5 and *S* = 1.25. The analysis was run in triplicate for 500 million generations with a sample frequency of 50,000 and 10% burn-in. Convergence of MCMC chains was assessed by visual examination of the log trace of each posterior distribution showing caterpillar shape in TRACER, and making sure that the Effective Sample Size (ESS) value of each statistic was >200 (Ho & Shapiro, 2011). The three runs were combined using LOGCOMBINER 2.1.3 (part of the BEAST package) and the final ESSs were at least 1,100. The estimate of *μ* was provided under the “*Estimates*” tab in TRACER as the mean of the “*clockRate*” parameter.

### Demography, selection and effective population size

Departure from mutation-drift equilibrium indicative of demographic change or selective sweep was assessed in dataset 1 using Tajima’s D (Tajima, 1989) and Fu’s Fs (Fu, 1997) tests implemented in ARLEQUIN and 10,000 coalescent-based simulations were run to calculate *p*-values. Since Tajima’s D and Fu’s Fs statistics are sensitive to both demographic change and selection, we also applied Fay & Wu’s H statistic (Fay & Wu, 2000) to dataset 1 for the single 16S, COI, Cyt*b* fragments and the concatenated 16S-COI-Cyt*b* data using DnaSP to attempt to discriminate between the effects of population size change and selection (Zeng et al., 2006). *Tectarius striatus* was the most closely related species to *M. neritoides* (Reid, Dyal & Williams, 2012) for which the three same gene fragments of 16S, COI and Cyt*b* were available on Genbank (U46825, AJ488644, U46826), and was therefore used as outgroup for the Fay & Wu test. Confidence intervals were calculated based on 10,000 coalescent-based simulations.

ARLEQUIN was used to construct a distribution of pairwise nucleotide differences between haplotypes (sequence mismatch distribution) and to compare this distribution with the expectations of a sudden expansion model (Harpending, 1994; Li, 1977; Rogers, 1995). Although the analysis complied with the assumption of panmixis, it did not do so with respect to neutrality (see ‘Results’), thus limiting the reliability of the results. Three demographic parameters were inferred using a generalized nonlinear least-squares method to determine whether *M. neritoides* has undergone sudden population growth: the rate of population growth *τ* = 2μ*t* (*t* being the time since the expansion), the initial population size before the growth (*θ*_0_), and the final population size after growth (*θ*_1_). The goodness of fit between the observed and expected mismatch distributions was tested by parametric bootstrapping of the sum of squared deviations (Ssd) (Schneider & Excoffier, 1999), and by the Harpending Raggedness index (r) (Harpending et al., 1998).

A time-calibrated Bayesian skyline plot (BSP) was built for dataset 1 using BEAST, to detect past population dynamics through time and to estimate *N*_*e*_ of *M. neritoides* in the Azores. The coalescent priors used in the skyline plot model assume a random sample of orthologous, non-recombining and neutrally evolving sequences from a panmictic population. The skyline plot model has been shown to be robust to violation of these assumptions and to correctly reconstruct demographic history with mtDNA (Drummond et al., 2005). However, recent studies show that violation of these assumptions may still affect the estimated population size variation, and that the BSP is prone to confound the effect of population structure with declines in population size in panmictic populations, or fails to detect population expansion (Grant, 2015; Heller, Chikhi & Siegismund, 2013). Hence, population structure and selection were assessed beforehand using Tajima’s D, Fu’s Fs and Fay & Wu’s H statistics. The BSP analyses were performed under a HKY substitution model with empirical base frequencies, a fixed substitution rate equal to 1.0, and a piecewise-constant Bayesian skyline model with 10 groups. The prior on the clockRate parameter was set to a log-normal with *M* = − 5 and *S* = 1.25. Analyses were run in triplicate for 200 million generations with a sample frequency of 20,000 and 10% burn-in. After combination of the three runs, the final ESSs were at least 1,000. *N*_*e*_ was extracted from the BSP, by dividing the median value of the ${N}_{e}^{\ast }\tau $ product in the most recent year 1996 (${N}_{e}^{\ast }\tau \approx \text{17,977}$) by the generation time *τ* = 3 years and five months (Hughes & Roberts, 1981).

## Results

### mtDNA diversity of *Melarhaphe neritoides*

Dataset 1, representing the overall population of the Azores archipelago (*N* = 185 from five localities in the archipelago), contains 184 different and private haplotypes (*H* = 184; *H*_*p*_ = 184 and hence *H*_*s*_ = 0) (Table 1), except for one haplotype that was found in two individuals from Pico island (*H*_*w*_ = 1). Hence, the frequency of this latter, i.e., the most common, haplotype was 0.0108, while all other haplotypes had a frequency of 0.00541. This remarkable mtDNA diversity is further reflected by a haplotype diversity (based on the concatenated 16S-COI-Cyt*b* data) close to its maximum value 1 (*Hd* = 0.999 ± 0.001), indicating a probability of less than 0.001% that two individuals from the same locality share the same haplotype in the overall population of the archipelago. One fourth of the 1771 nucleotide positions are polymorphic (*S* = 23.7%) with 167 sites (9.4%) showing one variant, 225 sites (12.7%) two variants, 24 sites (1.4%) three variants, and four sites (0.2%) four variants. Moreover, there is on average 1.3% nucleotide differences per site between two randomly chosen DNA sequences in the overall population (*π* = 0.013 ± 0.001). More precisely, among the protein-coding COI and Cyt*b* regions (1,289 bp), we observed 323 (18%) sites with synonymous and 964 (54%) sites with non-synonymous substitutions, yielding on average *π*_syn_ = 0.0677(6.77%) and *π*_non−syn_ = 0.0004(0.04%) at synonymous and non-synonymous sites respectively. Repeating COI and 16S PCR amplifications on 20 specimens yielded identical sequence results, confirming that PCR did not generate artificial variation. For 185 query sequences of *M. neritoides* submitted to BOLD, that stores 51 barcodes of *M. neritoides* (data retrieved from BOLD on 13 October 2015), the identification engine returns 100% correct identifications under one and the same Barcode Index Number (BIN = BOLD:AAG4377).

**Figure 2:**
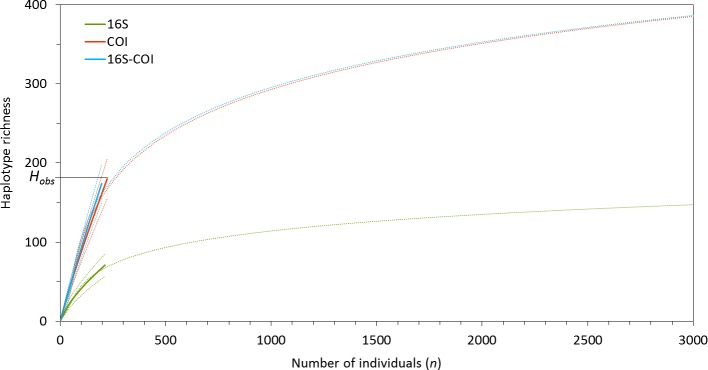
Individual-based rarefaction curves (solid lines) and 95% confidence intervals (dashed lines) based on COI, 16S and concatenated 16S-COI data, based on *M. neritoides* specimens sampled in Mosteiros (MOS), São Miguel island. *H*_obs_ is the haplotype richness observed in the actual sample (*n*) from MOS. The logarithmic trendlines (dotted lines) show a prediction of the haplotype richness expected for larger sampling size at the MOS sampling site.

**Figure 3:**
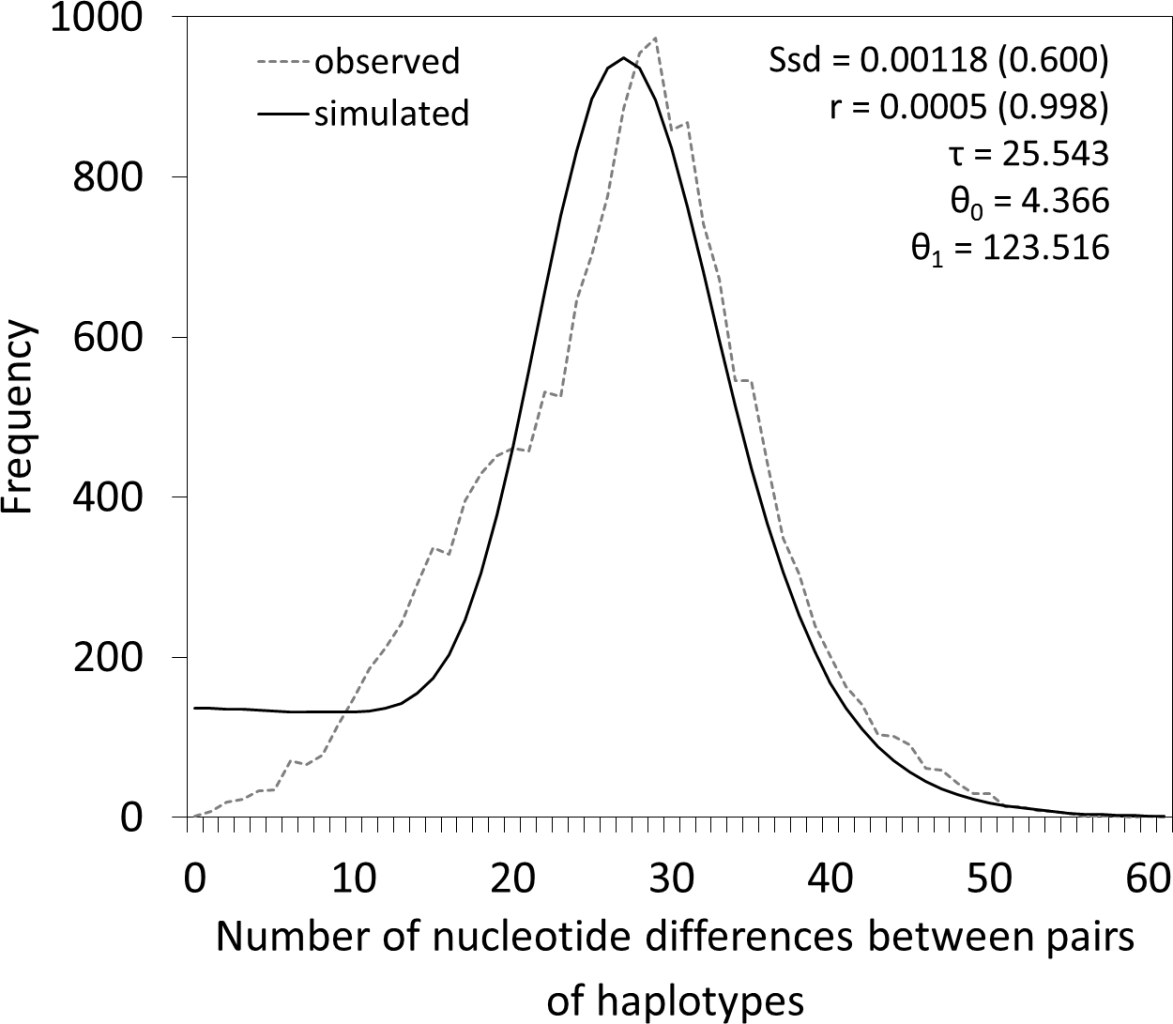
Mismatch distribution analysis showing the unimodal distribution of the observed number of differences between pairs of haplotypes of *M. neritoides*. Ssd, sum of squared differences and *p*-value in parenthesis; *r*, Harpending’s Raggedness index and *p*-value in parenthesis; *τ*, time in generations since the last demographic expansion; *θ*_0_, initial population size; *θ*_1_, final population size.

The individual-based rarefaction curves of 16S, COI and 16S-COI (dataset 2) do not reach a plateau, but their steep slopes decrease according to 16S-COI > COI > 16S (Fig. 2). *H*_obs_ values are close to the maximal sampling size *n* for the COI (*H*_obs_ = 180, *n* = 223) and the 16S-COI (*H*_obs_ = 174, *n* = 197) fragments indicating that a large fraction of the haplotype diversity remains to be discovered, whereas it is further from *n* for the 16S fragment (*H*_obs_ = 71, *n* = 213). The logarithmic trendlines representative of the population growth in the species show inflexion around large sampling sizes (*n* > 500), indicating that additional sampling is likely to yield new haplotypes. Indeed, the *Chao1* (*chao1 mean* = 1596.22, CI = [878.94–3043.34], *n* = 197) and *Chao2* (*chao2 mean* = 1486.20, CI = [842.91–2748.15], *n* = 197) estimators for the concatenated 16S-COI gene fragment suggest that the predicted total haplotype richness of *M. neritoides* would be reached by sampling 1,500 individuals per sampling site.

### Demography, selection and mutation rate

**Figure 4:**
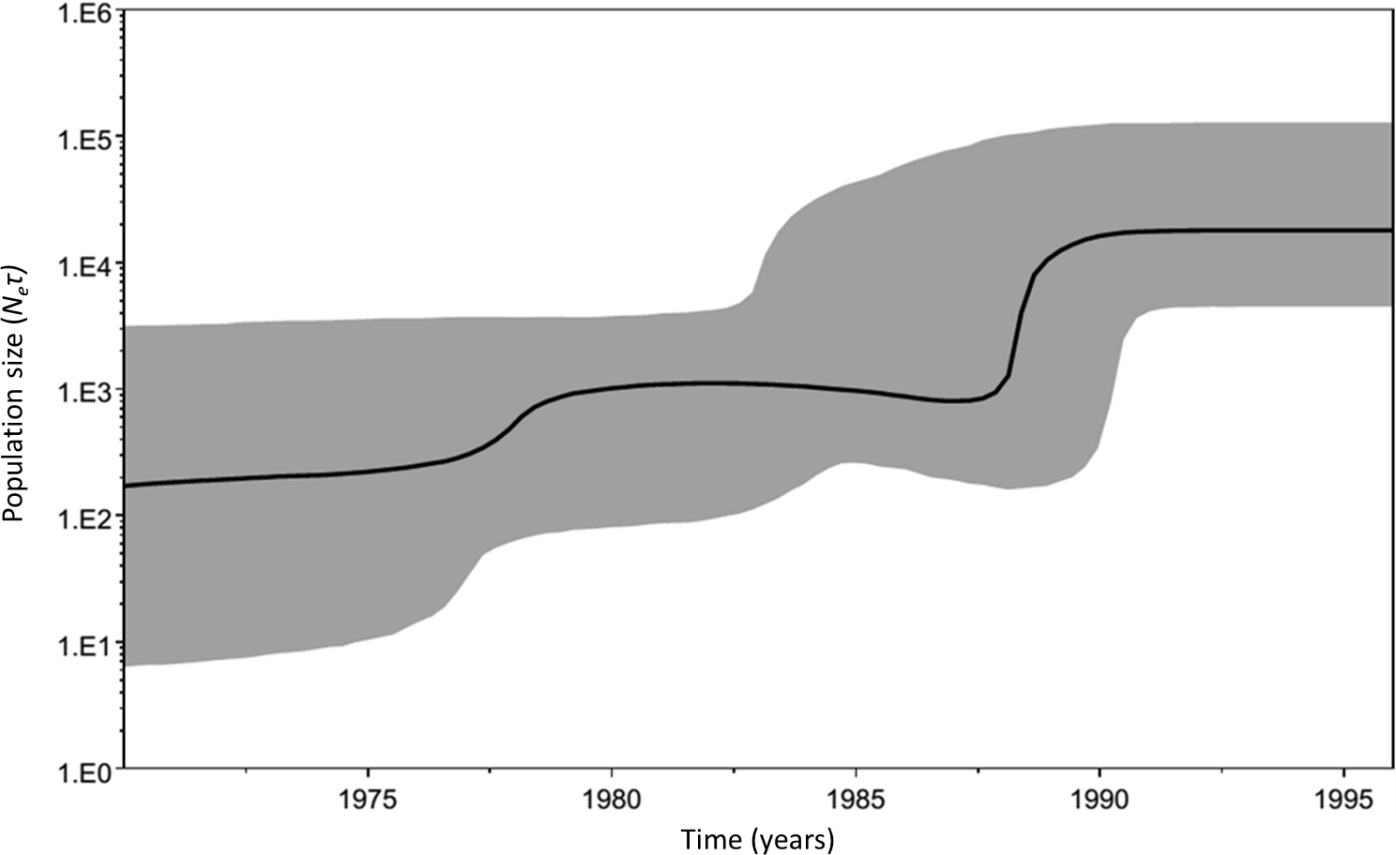
Historical demographic trends of the median estimate of the maternal effective population size over time (bold line) constructed using a Bayesian skyline plot approach based on concatenated 16S-COI-Cyt *b* haplotypes of *M. neritoides* sampled in 1992, 1993 and 1996. The *y*-axis is the product of effective population size (*N*_*e*_) and generation time (*τ*) in a log scale, while the *x*-axis is a linear scale of time in years. The 95% highest probability density (HPD) intervals are shaded in grey and represent both phylogenetic and coalescent uncertainty.

Both Tajima’s D and Fu’s Fs tests show a significant departure of *M. neritoides* from constant population size or neutrality (*D* = − 2.030, *p* < 0.01 and *Fs* = − 23.706, *p* < 0.01), suggesting demographic expansion and/or a potential action of selection. Fay & Wu’s H, which is sensitive to positive selection and not to population growth or background selection, shows significant signal of selection for 16S (*H* = − 30.42, CI = [−3.06–1.13]), COI (*H* = − 85.16, CI = [−17.05–6.08]), Cyt*b* (*H* = − 110.38, CI = [−13.33–5.18]) and the concatenated 16S-COI-Cyt*b* fragment (*H* = − 225.96, CI = [−30.36–11.22]). The unimodal curve of the sequence mismatch distribution (Fig. 3) suggests that population expansion cannot be rejected as *θ*_0_ < *θ*_1_ (*τ* = 25.543, *θ*_0_ = 4.366, *θ*_1_ = 123.516). The non-significant values of the sum of squared deviations (*Ssd* = 0.00118, *p* = 0.600) and Harpending’s Raggedness index (*r* = 0.0005, *p* = 0.998) show that the sudden expansion model provides a good fit to the data. The time-calibrated BSP shows an increase of *N*_*e*_ through time, indicating that *M. neritoides* has been expanding in the Azores archipelago or has undergone selection (Fig. 4). For the year 1996, the BSP gives ${N}_{e}^{\ast }\tau \approx \text{17,977}$, corresponding to *N*_*e*_ ranging from 1,312 to 37,495 with an average *N*_*e*_ ≈ 5,256 individuals.

With data sampled in 1992, 1993 and 2012 (i.e., an interval of 20 years), we estimated a mutation rate of μ = 5.82 × 10^−5^ per nucleotide site per year at COI. Considering a generation time of *τ* = 41 months (i.e., 3.42 years), the mutation rate was estimated to be μ = 1.99 × 10^−4^ mutations per nucleotide site per generation.

### Population genetic structure

**Figure 5:**
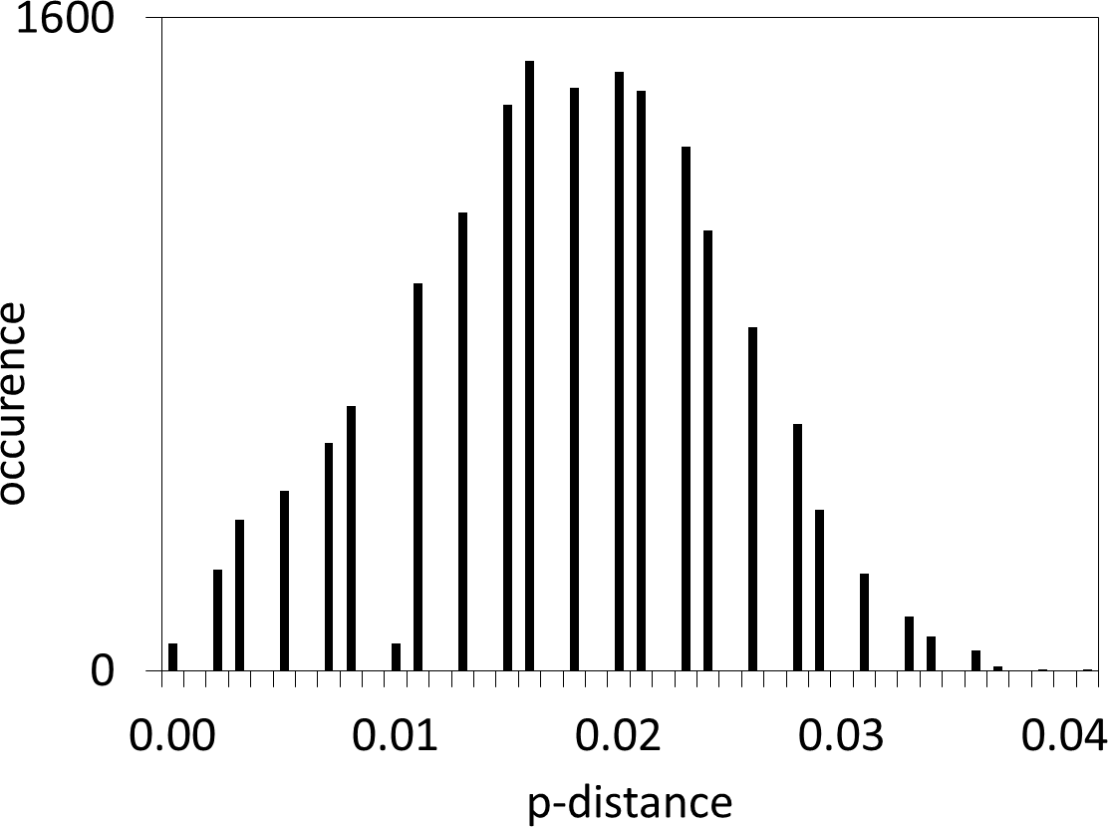
Distribution of COI pairwise p-distances in *M. neritoides*.

All phylogenetic trees provided maximal support for the monophyly of *M. neritoides* (trees not shown). Additionally, the three species delimitation methods, ABGD, bPTP and GMYC, lumped *M. neritoides* as one Molecular Operational Taxonomic Unit (trees not shown). The mean intraspecific p-distance within *M. neritoides* was *d* = 0.018 ± 0.002, i.e., one order of magnitude greater than the mean intraspecific p-distances of the three other species, *viz. Littorina littorea* (*d* = 0.004 ± 0.001), *Pomatias elegans* (*d* = 0.009 ± 0.002) and *Tectarius striatus* (*d* = 0.006 ± 0.001), but still far below interspecific p-distances ranging from 0.166 to 0.271 for the 36 possible species pairs of Littorinoidea, from 0.166 to 0.246 for the 21 species pairs of Littorinidae, or from 0.187 to 0.225 for the six species pairs of Littorininae (Table S2). The Gaussian distribution of intraspecific COI p-distances in *M. neritoides* (Fig. 5) indicates that the five populations sampled on five different islands of the Azores archipelago form a homogeneous haplotype mixture without any evidence of a DNA barcode gap.

**Figure 6:**
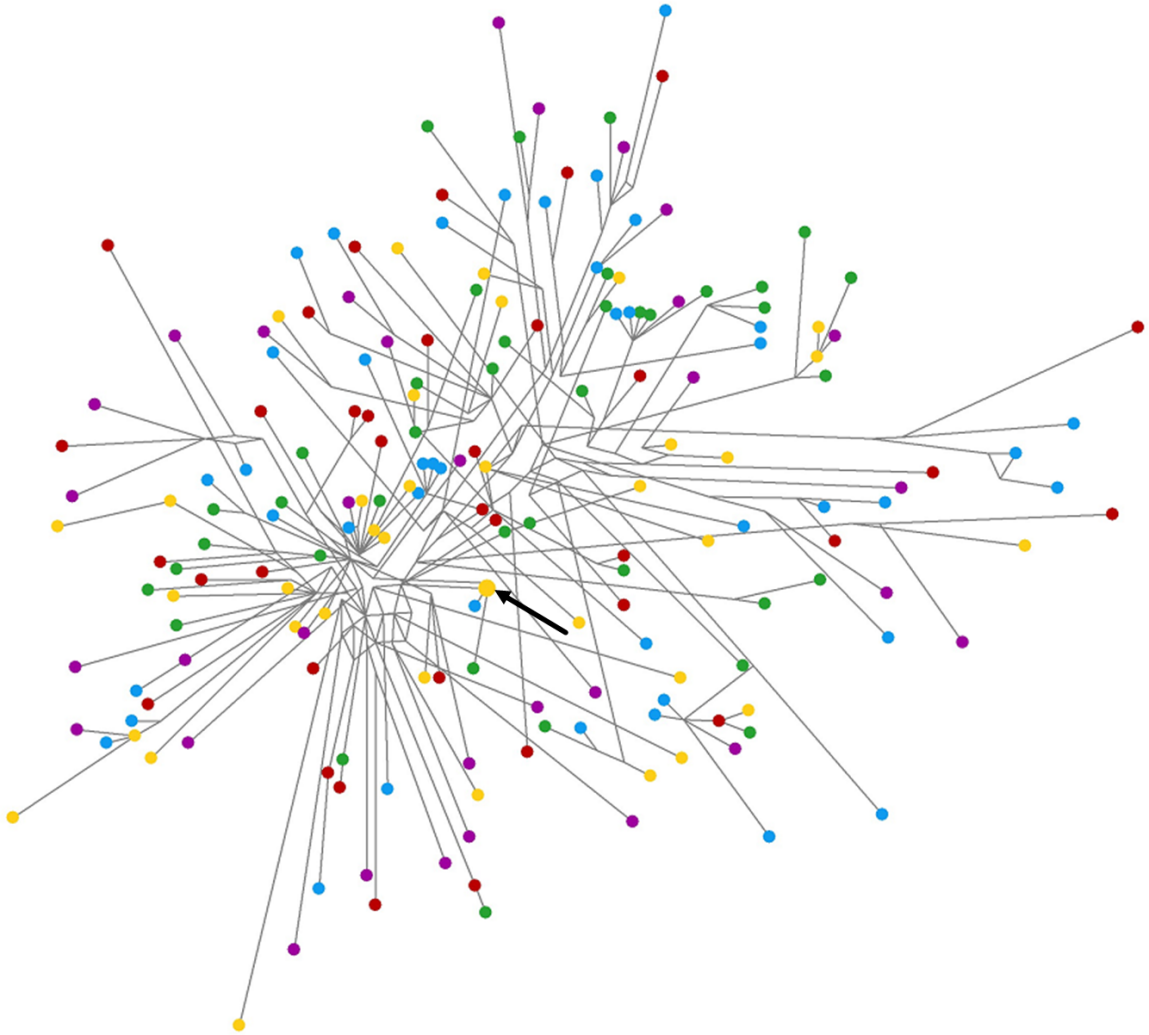
Median-joining network of mtDNA in *M. neritoides*. Branch lengths are proportional to the numbers of mutational steps separating haplotypes. The size of circles is proportional to the number of individuals per haplotype and the sole haplotype shared by two individuals is marked by an arrow. Haplotype origins: Flores island—green; Faial island—blue; Pico island—yellow; São Miguel island—red; Santa Maria island—purple.

The bush-like pattern of the mtDNA haplotype network (Fig. 6) shows the overwhelming number of unique, private haplotypes represented by single individuals (i.e., singletons), the lack of shared haplotypes between sites, and several homoplastic character states (cycles). The apparent lack of association between genetic variation and geographic location (as revealed by the distribution of colours across the network of Fig. 6) suggests the absence of phylogeographic structure in Azorean *M. neritoides*.

The low and non-significant indices of population genetic differentiation (*G*_ST_ = 0.0003, *p* = 0.1676; *N*_ST_ = 0.0021, *p* = 0.5346; φ_ST_ = 0.0026, *p* = 0.2220) make that the hypothesis of panmixis (and hence no population structuring) cannot be rejected.

## Discussion

### How diverse is the mtDNA of *Melarhaphe neritoides?*

Azorean *M. neritoides* harbours a remarkable amount of intraspecific mtDNA diversity, characterized by very high haplotype diversity and nucleotide diversity with respect to the concatenated 16S-COI-Cyt*b* gene fragments, at the single Cyt*b* gene fragment and at the single COI gene fragment. Moreover, it shows a value of neutral mtDNA nucleotide diversity *π*_syn_ ≥ the threshold of 5% for the concatenated 16S-COI-Cyt*b* fragments, and is therefore qualified as hyperpolymorphic. The *π*_syn_ values for COI and Cyt*b* separately are also ≥ 0.05 and support mtDNA hyperdiversity in *M. neritoides* (Table 1). mtDNA hyperdiversity is also observed in a Spanish population. The COI data retrieved from García et al. (2013) yielded *π*_syn_ = 0.0762 (7.62%) and *π*_non−syn_ = 0.0002 (0.02%) in a local Spanish population of 49 individuals. These values are very similar to those of COI in the Azorean populations (Table 1). Therefore, mtDNA hyperdiversity is not a local characteristic of *M. neritoides* along the Iberian Atlantic coast, but is shared more broadly in the Azorean populations, and presumably, throughout the species’ distribution range. The high *π* values in *M. neritoides* reflect natural variation, not PCR errors, as validated by the identical triplicates of mtDNA sequences and 100% correct species identification using barcoding. DNA barcoding is based on the premise that COI sequence divergence is higher among species than within species (Hebert, Ratnasingham & DeWaard, 2003), and might be hampered by high mtDNA variation, specifically COI hyperdiversity and high intraspecific sequence divergence in COI. Yet, in spite of the highly variable COI marker in *M. neritoides* (*π* = 0.018 ± 0.001) and elevated intraspecific p-distance (*d* = 0.001–0.041), the ability of DNA barcoding to identify *M. neritoides* is not affected by this mtDNA hyperdiversity.

The mtDNA of *M. neritoides* is more diverse than (1) mtDNA of most temperate littorinids and many tropical littorinids, (2) mtDNA of many planktonic-dispersing marine invertebrates, and (3) mtDNA of other hyperdiverse Mollusca (Table 2). More specifically, in comparison with 26 other littorinid species, *M. neritoides* has the highest COI haplotype diversity among temperate species (i.e., *Austrolittorina* spp., *Bembicium vittatum*, *Littorina* spp., *Tectarius striatus*) and the same degree as two tropical species *Echinolittorina reticulata* and *Echinolittorina vidua*. *Melarhaphe neritoides* also has the highest COI nucleotide diversity among temperate species, and shows a higher COI nucleotide diversity than tropical species (i.e., *Bembicium nanum*, *Cenchritis muricatus*, *Echinolittorina* spp., *Littoraria* spp.) except for *Echinolittorina vidua* whose nucleotide diversity (*π* = 0.041) is about twice that of *M. neritoides* (*π* = 0.018). In comparison to 15 other non-littorinid marine invertebrates with similar planktonic larval dispersal and high mtDNA variability, *M. neritoides* has the highest COI haplotype diversity. Yet, *M. neritoides* shows the same degree of COI haplotype diversity as the pelagic nudibranch *Glaucus atlanticus* (*Hd* = 0.996) and the annelid *Pygospio elegans* (*Hd* = 0.996). Regarding COI nucleotide diversity, *M. neritoides* has the highest value among annelids, arthropods, cnidarians, echinoderms, other gastropods, and some bivalves (but not all). Two bivalves, viz. *Brachidontes pharaonis* and *Tridacna maxima*, show very high COI nucleotide diversities that probably reflect ongoing speciation in the three lineages of the *Brachidontes spp.* complex (Terranova et al., 2007) and in the four lineages in *Tridacna maxima* (Nuryanto & Kochzius, 2009). The literature data in Table 2 suggest that there is no obvious correlation between *π* and *Hd*. However, more data are needed to corroborate this observation.

We estimated the neutral component of the COI nucleotide hyperdiversity in *M. neritoides*, i.e., *π*_syn_ = 0.074, on which the diagnosis of hyperdiversity is based. In comparison to eight other mollusc species with hyperdiverse mtDNA (Table 2), *M. neritoides* is situated in the lower part of the neutral nucleotide diversity range (*π*_*syn*_ = [0.066–0.256]).

### mtDNA divergence and population structuring in *Melarhaphe neritoides*

We investigated whether population genetic structure through time and space, and cryptic taxa, could contribute to the mtDNA hyperdiversity in *M. neritoides*. The monophyly of *M. neritoides* and the Gaussian distribution of its intraspecific p-distances, suggest that *M. neritoides* does not conceal cryptic taxa in the Azores. Conversely, the overwhelming number of private haplotypes (Fig. 6) at first glance suggest that populations are strongly differentiated because of the apparent lack of shared haplotypes. Yet, the bush-like mtDNA haplotype network (Fig. 6) is suggestive of complete population mixing (Nielsen & Slatkin, 2013). Indeed, recurrent long-term gene flow homogenising the gene pool of *M. neritoides* over the 600 km between the Azorean islands implies an absence of population genetic structure (differentiation), as is reflected in the *G*_ST_, *N*_ST_ and φ_ST_ values that are not significantly different from zero. This is congruent with the low level of differentiation and high potential for long range gene flow between Swedish and Cretan populations of *M. neritoides* (Johannesson, 1992). Currently, no other data on population genetic differentiation and gene flow in *M. neritoides* are available. The possibility of long-distance gene flow may suggest that the mtDNA diversity of *M. neritoides* in the Azores is the result of larval influx from European populations. Yet, while short-lived Pleistocene westward-flowing sea surface currents allowed the colonization of the Azores from Eastern Atlantic areas (Ávila et al., 2009), the eastward-flowing Azores Current nowadays (Barton, 2001) suggests that larval transport predominantly occurs from the Azores towards the North East Atlantic coasts and the Mediterranean Sea, and that the Azores rather may act as a source of new, dispersing, haplotypes than as a sink receiving new haplotypes. Hence, all current evidence suggests that mtDNA hyperdiversity in *M. neritoides* is not due to (1) population structuring, (2) admixture of divergent local populations, (3) lumping of cryptic taxa, or (4) influx of new haplotypes from distant European populations.

### mtDNA mutation rate in *Melarhaphe neritoides*

We investigated whether mtDNA mutation rate explains mtDNA hyperdiversity. The mutation rate is the rate at which new mutations arise in each generation of a species and accumulate per DNA sequence, and differs from the substitution rate that accounts for the fraction of new mutations that do not persist in the face of evolutionary forces (Barrick & Lenski, 2013). Accordingly, neutral synonymous mutations reflect the mutation rate (Barrick & Lenski, 2013). Mutation rates in most nuclear eukaryotic genomes are generally extremely low because elaborate molecular mechanisms correct errors in DNA replication and repair DNA damage, whereas viral and animal mitochondrial genomes have no, or far less efficient, repair mechanisms and thus have much higher mutation rates (Ballard & Whitlock, 2004; Drake et al., 1998). Overall, synonymous mutations become fixed at a rate that appears to be uniform across various taxa (Kondrashov, 2008), and mtDNA mutation rates lie in a narrow range of 10^−8^–10^−7^ mutations per nucleotide site per generation across e.g., arthropods, echinoderms, chordates, molluscs and nematodes (Table 3). Surprisingly, our estimate of the mtDNA COI mutation rate in *M. neritoides* (μ = 1.99 × 10^−4^ per site per generation) is 1,000 to 10,000-fold higher than commonly estimated for the mtDNA mutation rates in metazoans from these phyla. Therefore, if our inference is correct, it seems likely that this high mtDNA mutation rate substantially contributed to generating the mtDNA hyperdiversity in *M. neritoides*. Our mutation rate estimate was obtained from mtDNA sequence data of *M. neritoides* itself, not from closely related species, and is therefore expected to be more accurate and species-specific. Bayesian MCMC estimates of substitution rates based on heterochronous mtDNA samples may be susceptible to an upward bias when populations have a complex demographic history (e.g., bottleneck) or pronounced population structure. Hence, such biased estimates may reflect other processes like migration, selection and genetic drift rather than mutation (Navascués & Emerson, 2009). However, this study did not provide evidence of population structure in *M. neritoides*, reducing therefore the risk of bias in the estimate of *μ*. Bayesian MCMC inferences based on heterochronous mtDNA samples over short timescales may also overestimate generational mutation rates by an order of magnitude in comparison to phylogenetically derived mutation rates, because they may account for short-lived, slightly deleterious mutations at non-synonymous sites (Ho et al., 2005; Penny, 2005; Subramanian & Lambert, 2011). Since *μ* in *M. neritoides* was estimated over a short period of 20 years, it may be subject to such a bias. However, while this bias could have generated an order of magnitude overestimation of *μ*, it cannot entirely account for the extreme value inferred, which is 10^3^ to 10^4^ fold higher than usually estimated for other organisms (Subramanian & Lambert, 2011).

**Table 3:** mtDNA mutation rates per site per generation in various metazoans ranked according to decreasing *μ*.

Species		*μ*	locus	Reference
*Melarhaphe neritoides*	Mo	1.99 × 10^−4^	COI	this study
*Homo sapiens sapiens*	Ch	6.00 × 10^−7^	mt genome	Kivisild (2015)
*Caenorhabditis elegans*	Ne	1.60 × 10^−7^	mt genome	Denver et al. (2000)
*Mytilus edulis*	Mo	9.51 × 10^−8^	COI	Wares & Cunningham (2001)
*Drosophila melanogaster*	Ar	6.20 × 10^−8^	mt genome	Haag-Liautard et al. (2008)
*Asteria rubens*	Ec	4.84 × 10^−8^	COI	Wares & Cunningham (2001)
*Nucella lapillus*	Mo	4.43 × 10^−8^	COI	Wares & Cunningham (2001)
*Euraphia* spp.	Ar	3.80 × 10^−8^	COI	Wares & Cunningham (2001)
*Idotea balthica*	Ar	3.60 × 10^−8^	COI	Wares & Cunningham (2001)
*Semibalanus balanoides*	Ar	2.76 × 10^−8^	COI	Wares & Cunningham (2001)
*Littorina obtusata*	Mo	2.49 × 10^−8^	COI	Wares & Cunningham (2001)
*Sesarma* spp.	Ar	2.10 × 10^−8^	COI	Wares & Cunningham (2001)
*Alpheus* spp.	Ar	1.90 × 10^−8^	COI	Wares & Cunningham (2001)
*Prochilodus* spp.	Ch	0.27 × 10^−8^	COI	Turner et al. (2004)

**Notes.**

Ar Arthropoda Ch Chordata Ec Echinodermata Mo Mollusca Ne Nematoda

Invertebrates with shorter generation times have higher mtDNA mutation rates, as their mitochondrial genomes are copied more frequently (Thomas et al., 2010). In comparison to the generation times of invertebrates analyzed by Thomas et al. (2010), ranging from 8 days in the hydrozoan *Hydra magnipapillata* to 1,825 days in the coral *Montastraea annularis* and the seastar *Pisaster ochraceus*, the generation time of *M. neritoides* (*τ* ≈ 1,250 days) is not particularly short and therefore its mtDNA mutation rate would be expected to be at the lower side. Yet, *M. neritoides* has a high mtDNA mutation rate (μ = 5.82 × 10^−5^ per site per year) that does not fall within the range of mutation rates of invertebrates with longer generation times than *M. neritoides*, i.e., from μ = 3 × 10^−10^ per site per year in *Montastraea annularis* (Fukami & Knowlton, 2005) to μ = 2.81 × 10^−6^ per gene per year in *Pisaster ochraceus* (Popovic et al., 2014).

High mtDNA mutation rates may be more frequently linked to hyperdiversity than previously thought in the widely used COI marker. Indeed, neutral nucleotide diversities of ≥0.05 have been reported in 222 other species among Arthropoda, Chordata, Echinodermata, Mollusca and Nematoda (Table S1), suggesting the possibility of underlying high mtDNA mutation rates.

### Demography and selection in *Melarhaphe neritoides*

We investigated whether selection, mtDNA demographic history and *N*_*e*_ explain mtDNA hyperdiversity. Equilibrium between variation gained by mutations and variation lost by genetic drift should be reached if the effective population size has been stable over time and in absence of population structure or selection (Kimura, 1983). According to the negative Tajima’s D, Fu’s Fs and Fay & Wu’s H, the unimodal sequence mismatch distribution and the BSP trend, the phylogeny of Azorean *M. neritoides* has been shaped either by demographic expansion or selection, or a combination of both.

The effective mtDNA population size of *M. neritoides* estimated in this paper is *N*_*e*_ ≈ 5, 256 (CI = 1,312–37,495) for the concatenated 16S-COI-Cyt*b* gene fragments. This is relatively small in comparison to mtDNA *N*_*e*_ of other littorinids with planktonic larval stages and high dispersal potential like *Littorina plena* (*N*_*e*_ = 16,0526–33,728,571) and *Littorina scutulata* (*N*_*e*_ = 90,790–3,814,286) (Table 4), except for the mtDNA *N*_*e*_ in the planktonic dispersing *Littorina keenae* (*N*_*e*_ = 135) (Lee & Boulding, 2007). Yet, this latter value refers to one sampling site only, whereas another sampling site of *Littorina keenae* showed a much larger mtDNA *N*_*e*_ (*N*_*e*_ = 31,797). Surprisingly, and somewhat counterintuitively, the mtDNA of *M. neritoides* is also smaller than that of periwinkles without planktonic larval stages, such as *Littorina sitkana* (*N*_*e*_ = 105,263–1,400,000) and *Littorina subrotundata* (*N*_*e*_ = 25,000 –1,942,857) (Lee & Boulding, 2009). However, past putative selection in *M. neritoides* likely confounds the BSP inference by reducing the overall mtDNA diversity and thus the mtDNA *N*_*e*_ estimate. As such, mtDNA variation in *M. neritoides* is still remarkably high, despite this signal of a reduction of its diversity by the putative influence of selection. This strengthens the hypothesis that the mtDNA hyperdiversity in *M. neritoides* is best explained by a high *μ* of mtDNA.

**Table 4:** mtDNA effective population sizes (*N*_*e*_) for various taxa. The 95% confidence interval is given in parenthesis when available.

Taxon		*N*_*e*_	Locus	Reference
*Littorina keenae*	Mo	135 (42–2,490)	ND6-Cyt*b*	Lee & Boulding (2007)
*Melarhaphe neritoides*	Mo	5,256 (1,312–37,495)	COI-16S-Cyt*b*	this study
*Homo* & *Pan*	Ch	5,900–10,000	mt genome	Piganeau & Eyre-Walker (2009)
Felidae & Canidae	Ch	130,000–430,000	mt genome	Piganeau & Eyre-Walker (2009)
*Pachygrapsus crassipes*	Ar	167,000–1,020,000	COI	Cassone & Boulding (2006)
*Cardinalis cardinalis*	Ch	193,000 (4,000–701,000)	ND2-Cyt*b*	Smith & Klicka (2013)
Murinae	Ch	230,000–730,000	mt genome	Piganeau & Eyre-Walker (2009)
*Littorina sitkana*	Mo	105,263–1,400,000	Cyt*b*	Lee & Boulding (2009)
*Littorina subrotundata*	Mo	25,000–1,942,857	Cyt*b*	Lee & Boulding (2009)
*Littorina scutulata*	Mo	90,790–3,814,286	Cyt*b*	Lee & Boulding (2009)
*Littorina plena*	Mo	160,526–33,728,571	Cyt*b*	Lee & Boulding (2009)

**Notes.**

Ar Arthropoda Ch Chordata Mo Mollusca

mtDNA *N*_*e*_ and mtDNA hyperdiversity may be positively correlated such as in the lined shore crab *Pachygrapsus crassipes* (*N*_*e*_ = 167,000–1,020,000; COI *Hd* = 0.923; *π* = 0.009) (Cassone & Boulding, 2006). Yet, this relationship has been questioned (Bazin, Glémin & Galtier, 2006; Piganeau & Eyre-Walker, 2009), because Bazin, Glémin & Galtier (2006) showed that mtDNA diversity is not linked to mtDNA *N*_*e*_, but rather to *μ* and selection. Conversely, Nabholz, Glémin & Galtier (2009) and Nabholz et al. (2008) found no link between selection and mtDNA *N*_*e*_, but confirmed that mtDNA diversity is strongly linked to *μ*. Our present work shows a link between mtDNA hyperdiversity and high mtDNA *μ*, and the putative influence of selection on *N*_*e*_ estimation making mtDNA *N*_*e*_ a poor indicator of mtDNA hyperdiversity.

## Conclusions

The mtDNA hyperdiversity of *M. neritoides* is characterized by a high haplotype diversity (*Hd* = 0.999 ± 0.001), a high nucleotide diversity (*π* = 0.013 ± 0.001) and a high neutral nucleotide diversity (*π*_neu_ = 0.0678) for the concatenated 16S-COI-Cyt*b* gene fragments. The mutation rate at the COI locus is μ = 1.99 × 10^−4^ mutations per nucleotide site per generation, which is a very high value. Demographic analyses revealed that *M. neritoides* in the Azores underwent a population expansion, but the effective population size *N*_*e*_ was surprisingly small for a planktonic-developing species (*N*_*e*_ = 5, 256; CI = 1,312–37,495) probably due to the putative influence of selection on *M. neritoides* mtDNA. As a result, *N*_*e*_ is not linked to mtDNA hyperdiversity and is a poor indicator of this latter. Mitochondrial DNA hyperdiversity is best explained by a high mtDNA *μ* in *M. neritoides*. Mitochondrial DNA hyperdiversity may be more common across eukaryotes than currently known.

##  Supplemental Information

10.7717/peerj.2549/supp-1 Table S1 List of 215 animal species with hyperdiverse DNA (*π*_syn_ > 0.05)

10.7717/peerj.2549/supp-2 Table S2 Specimen samples and data sets used in this study *N*, number of individuals; *N1*, *N2*, *N3*, *N4*, number of individuals used in dataset 1, dataset 2, dataset 3 and dataset 4 respectively.

10.7717/peerj.2549/supp-3 Table S3 Estimates of average evolutionary divergence over COI sequence pairs within and between groups n, number of sequences used; *d*_1_, number of base differences per site (p-distance) from averaging over all sequence pairs within each group ± standard error; n/c, cases in which it was not possible to estimate evolutionary distances; *d*_2_, number of base differences per site (p-distance) from averaging over all sequence pairs between groups (under diagonal) and standard error estimates (above diagonal).

## Additional Information and Declarations

### DNA Deposition

The following information was supplied regarding the deposition of DNA sequences: New sequences are available from Genbank with accession numbers from KT996151 to KT997344.

### Data Availability

The following information was supplied regarding data availability: Dataset 1: https://dx.doi.org/10.6084/m9.figshare.3437333.v4; Dataset 2 (16S): https://dx.doi.org/10.6084/m9.figshare.3437390.v2; Dataset 2 (COI): https://dx.doi.org/10.6084/m9.figshare.3437474.v2; Dataset 2 (16S_COI): https://dx.doi.org/10.6084/m9.figshare.3437489.v2; Dataset 3: https://dx.doi.org/10.6084/m9.figshare.3437498.v2; Dataset 4: https://dx.doi.org/10.6084/m9.figshare.3437531.v2.

## References

[ref-1] Anisimova M, Liberles DA, Cannarozzi GM, Schneider A (2012). Detecting and understanding natural selection. Codon evolution: mechanisms and models.

[ref-2] Aranzamendi MC, Bastida R, Gardenal CN (2011). Different evolutionary histories in two sympatric limpets of the genus *Nacella* (Patellogastropoda) in the South-western Atlantic coast. Marine Biology.

[ref-3] Ávila SP, Marques Da Silva C, Schiebel R, Cecca F, Backeljau T, De Frias Martins AM (2009). How did they get here? The biogeography of the marine molluscs of the Azores. Bulletin de la Société Géologique de France.

[ref-4] Ayre DJ, Minchinton TE, Perrin C (2009). Does life history predict past and current connectivity for rocky intertidal invertebrates across a marine biogeographic barrier?. Molecular Ecology.

[ref-5] Ballard JWO, Whitlock MC (2004). The incomplete natural history of mitochondria. Molecular Ecology.

[ref-6] Bandelt HJ, Forster P, Röhl A (1999). Median-joining networks for inferring intraspecific phylogenies. Molecular Biology and Evolution.

[ref-7] Barrick JE, Lenski RE (2013). Genome dynamics during experimental evolution. Nature Reviews Genetics.

[ref-8] Barton ED (2001). Canary and Portugal currents. Encyclopedia of ocean sciences.

[ref-9] Bazin E, Glémin S, Galtier N (2006). Population size does not influence mitochondrial genetic diversity in animals. Science.

[ref-10] Bird CE, Holland BS, Bowen BW, Toonen RJ (2007). Contrasting phylogeography in three endemic Hawaiian limpets (*Cellana* spp.) with similar life histories. Molecular Ecology.

[ref-11] Blakeslee AMH, Byers JE, Lesser MP (2008). Solving cryptogenic histories using host and parasite molecular genetics: the resolution of *Littorina littorea*’s North American origin. Molecular Ecology.

[ref-12] Blier PU, Dufresne F, Burton RS (2001). Natural selection and the evolution of mtDNA-encoded peptides: evidence for intergenomic co-adaptation. Trends in Genetics.

[ref-13] Bouckaert R, Heled J, Kuehnert D, Vaughan T, Wu C-H, Xie D, Suchard MA, Rambaut A, Drummond AJ (2014). BEAST 2: a software platform for Bayesian evolutionary analysis. PLoS Computational Biology.

[ref-14] Burton RS, Barreto FS (2012). A disproportionate role for mtDNA in Dobzhansky–Muller incompatibilities?. Molecular Ecology.

[ref-15] Cassone BJ, Boulding EG (2006). Genetic structure and phylogeography of the lined shore crab, *Pachygrapsus crassipes*, along the northeastern and western Pacific coasts. Marine Biology.

[ref-16] Castelin M, Lorion J, Brisset J, Cruaud C, Maestrati P, Utge J, Samadi S (2012). Speciation patterns in gastropods with long-lived larvae from deep-sea seamounts. Molecular Ecology.

[ref-17] Chao A (1984). Nonparametric estimation of the number of classes in a population. Scandinavian Journal of Statistics.

[ref-18] Chao A (1987). Estimating the population size for capture-recapture data with unequal catchability. Biometrics.

[ref-19] Churchill CKC, Alejandrino A, Valdés Á, Ó Foighil D (2013). Parallel changes in genital morphology delineate cryptic diversification of planktonic nudibranchs. Proceedings of the Royal Society of London Series B: Biological Sciences.

[ref-20] Churchill CKC, Valdés Á, Ó Foighil D (2014). Afro-Eurasia and the Americas present barriers to gene flow for the cosmopolitan neustonic nudibranch *Glaucus atlanticus*. Marine Biology.

[ref-21] Colgan DJ, Ponder WF, Beacham E, Macaranas JM (2003). Gastropod phylogeny based on six segments from four genes representing coding or non-coding and mitochondrial or nuclear DNA. Molluscan Research.

[ref-22] Colwell RK (2006). http://purl.oclc.org/estimates.

[ref-23] Cronin MA, Myers AA, O’Riordan RM (2000). The reproductive cycle of the intertidal gastropod *Melarhaphe neritoides* on the west and south coasts of Ireland. Proceedings of the Royal Irish Academy Section B: Biological, Geological and Chemical Science.

[ref-24] Cutter AD, Baird SE, Charlesworth D (2006). High nucleotide polymorphism and rapid decay of linkage disequilibrium in wild populations of *Caenorhabditis remanei*. Genetics.

[ref-25] Cutter AD, Jovelin R, Dey A (2013). Molecular hyperdiversity and evolution in very large populations. Molecular Ecology.

[ref-26] Darriba D, Taboada GL, Doallo R, Posada D (2012). jModelTest 2: more models, new heuristics and parallel computing. Nature Methods.

[ref-27] Denver DR, Morris K, Lynch M, Vassilieva LL, Thomas WK (2000). High direct estimate of the mutation rate in the mitochondrial genome of *Caenorhabditis elegans*. Science.

[ref-28] Dey A, Chan CKW, Thomas CG, Cutter AD (2013). Molecular hyperdiversity defines populations of the nematode *Caenorhabditis brenneri*. Proceedings of the National Academy of Sciences of the United States of America.

[ref-29] Díaz-Ferguson E, Haney RA, Wares JP, Silliman BR (2012). Genetic structure and connectivity patterns of two Caribbean rocky intertidal gastropods. Journal of Molluscan Studies.

[ref-30] Díaz-Ferguson E, Robinson JD, Silliman B, Wares JP (2010). Comparative phylogeography of North American Atlantic salt marsh communities. Estuaries and Coasts.

[ref-31] Doellman MM, Trussell GC, Grahame JW, Vollmer SV (2011). Phylogeographic analysis reveals a deep lineage split within North Atlantic *Littorina saxatilis*. Proceedings of the Royal Society of London Series B: Biological Sciences.

[ref-32] Drake JW, Charlesworth B, Charlesworth D, Crow JF (1998). Rates of spontaneous mutation. Genetics.

[ref-33] Drummond AJ, Nicholls GK, Rodrigo AG, Solomon W (2002). Estimating mutation parameters, population history and genealogy simultaneously from temporally spaced sequence data. Genetics.

[ref-34] Drummond AJ, Pybus OG, Rambaut A, Forsberg R, Rodrigo AG (2003). Measurably evolving populations. Trends in Ecology & Evolution.

[ref-35] Drummond AJ, Rambaut A, Shapiro B, Pybus OG (2005). Bayesian coalescent inference of past population dynamics from molecular sequences. Molecular Biology and Evolution.

[ref-36] Eo SH, DeWoody JA (2010). Evolutionary rates of mitochondrial genomes correspond to diversification rates and to contemporary species richness in birds and reptiles. Proceedings of the Royal Society of London B: Biological Sciences.

[ref-37] Excoffier L, Lischer HEL (2010). Arlequin suite ver 3.5: a new series of programs to perform population genetics analyses under Linux and Windows. Molecular Ecology Resources.

[ref-38] Excoffier L, Smouse PE, Quattro JM (1992). Analysis of molecular variance inferred from metric distances among DNA haplotypes: application to human mitochondrial DNA restriction data. Genetics.

[ref-39] Fay JC, Wu C-I (2000). Hitchhiking under positive Darwinian selection. Genetics.

[ref-40] Féral J-P (2002). How useful are the genetic markers in attempts to understand and manage marine biodiversity?. Journal of Experimental Marine Biology and Ecology.

[ref-41] Folmer O, Black M, Hoeh W, Lutz R, Vrijenhoek R (1994). DNA primers for amplification of mitochondrial cytochrome c oxidase subunit I from diverse metazoan invertebrates. Molecular Marine Biology and Biotechnology.

[ref-42] Fretter V, Graham A (1980). The prosobranch molluscs of Britain and Denmark: marine Littorinacea, Part 5. Journal of Molluscan Studies.

[ref-43] Fretter V, Manly R (1977). The settlement and early benthic life of *Littorina neritoides* (L.) at Wembury, S. Devon. Journal of Molluscan Studies.

[ref-44] Fu Y-X (1997). Statistical tests of neutrality of mutations against population growth, hitchhiking and background selection. Genetics.

[ref-45] Fujisawa T, Barraclough TG (2013). Delimiting species using single-locus data and the Generalized Mixed Yule Coalescent approach: a revised method and evaluation on simulated data sets. Systematic Biology.

[ref-46] Fukami H, Knowlton N (2005). Analysis of complete mitochondrial DNA sequences of three members of the *Montastraea annularis* coral species complex (Cnidaria, Anthozoa, Scleractinia). Coral Reefs.

[ref-47] García SD, Diz AP, Sá-Pinto A, Rolán-Alvarez E (2013). Proteomic and morphological divergence in micro-allopatric morphotypes of *Melarhaphe neritoides* in the absence of genetic differentiation. Marine Ecology Progress Series.

[ref-48] Giribet G, Okusu A, Lindgren AR, Huff SW, Schrödl M, Nishiguchi MK (2006). Evidence for a clade composed of molluscs with serially repeated structures: Monoplacophorans are related to chitons. Proceedings of the National Academy of Sciences of the United States of America.

[ref-49] Glez-Peña D, Gómez-Blanco D, Reboiro-Jato M, Fdez-Riverola F, Posada D (2010). ALTER: program-oriented conversion of DNA and protein alignments. Nucleic Acids Research.

[ref-50] Grant WS (2015). Problems and cautions with sequence mismatch analysis and Bayesian skyline plots to infer historical demography. Journal of Heredity.

[ref-51] Haag-Liautard C, Coffey N, Houle D, Lynch M, Charlesworth B, Keightley PD (2008). Direct estimation of the mitochondrial DNA mutation rate in *Drosophila melanogaster*. PLoS Biology.

[ref-52] Hague MTJ, Routman EJ (2016). Does population size affect genetic diversity? A test with sympatric lizard species. Heredity.

[ref-53] Hall TA (1999). BioEdit: a user-friendly biological sequence alignment editor and analysis program for Windows 95/98/NT. Nucleic Acids Symposium Series.

[ref-54] Hardy OJ, Vekemans X (2002). SPAGeDI: a versatile computer program to analyse spatial genetic structure at the individual or population levels. Molecular Ecology Notes.

[ref-55] Harpending HC (1994). Signature of ancient population growth in a low-resolution mitochondrial DNA mismatch distribution. Human Biology.

[ref-56] Harpending HC, Batzer MA, Gurven M, Jorde LB, Rogers AR, Sherry ST (1998). Genetic traces of ancient demography. Proceedings of the National Academy of Sciences of the United States of America.

[ref-57] Hebert PDN, Ratnasingham S, DeWaard JR (2003). Barcoding animal life: cytochrome c oxidase subunit 1 divergences among closely related species. Proceedings of the Royal Society of London B: Biological Sciences.

[ref-58] Heller R, Chikhi L, Siegismund HR (2013). The confounding effect of population structure on Bayesian skyline plot inferences of demographic history. PLoS ONE.

[ref-59] Hill GE (2016). Mitonuclear coevolution as the genesis of speciation and the mitochondrial DNA barcode gap. Ecology and Evolution.

[ref-60] Ho SYW, Phillips MJ, Cooper A, Drummond AJ (2005). Time dependency of molecular rate estimates and systematic overestimation of recent divergence times. Molecular Biology and Evolution.

[ref-61] Ho SYW, Shapiro B (2011). Skyline-plot methods for estimating demographic history from nucleotide sequences. Molecular Ecology Resources.

[ref-62] Hughes RN, Roberts DJ (1981). Comparative demography of *Littorina rudis*, *L. nigrolineata* and *L. neritoides* on three contrasted shores in North Wales. Journal of Animal Ecology.

[ref-63] James JE, Piganeau G, Eyre-Walker A (2016). The rate of adaptive evolution in animal mitochondria. Molecular Ecology.

[ref-64] Johannesson K (1992). Genetic variability and large scale differentiation in two species of littorinid gastropods with planktotrophic development, *Littorina littorea* (L.) and *Melarhaphe* (*Littorina*) *neritoides* (L.) (Prosobranchia: Littorinacea), with notes on a mass occurrence of *M. neritoides* in Sweden. Biological Journal of the Linnean Society.

[ref-65] Johnson PLF, Slatkin M (2008). Accounting for bias from sequencing error in population genetic estimates. Molecular Biology and Evolution.

[ref-66] Kearse M, Moir R, Wilson A, Stones-Havas S, Cheung M, Sturrock S, Buxton S, Cooper A, Markowitz S, Duran C, Thierer T, Ashton B, Meintjes P, Drummond A (2012). Geneious Basic: an integrated and extendable desktop software platform for the organization and analysis of sequence data. Bioinformatics.

[ref-67] Kennington WJ, Hevroy TH, Johnson MS (2012). Long-term genetic monitoring reveals contrasting changes in the genetic composition of newly established populations of the intertidal snail *Bembicium vittatum*. Molecular Ecology.

[ref-68] Kesäniemi JE, Geuverink E, Knott KE (2012). Polymorphism in developmental mode and its effect on population genetic structure of a spionid polychaete, *Pygospio elegans*. Integrative and Comparative Biology.

[ref-69] Kimura M (1983). The neutral theory of molecular evolution.

[ref-70] Kivisild T (2015). Maternal ancestry and population history from whole mitochondrial genomes. Investigative Genetics.

[ref-71] Kochzius M, Nuryanto A (2008). Strong genetic population structure in the boring giant clam, *Tridacna crocea*, across the Indo-Malay archipelago: implications related to evolutionary processes and connectivity. Molecular Ecology.

[ref-72] Kondrashov AS (2008). Another step toward quantifying spontaneous mutation. Proceedings of the National Academy of Sciences of the United States of America.

[ref-73] Lane N (2009). Biodiversity: on the origin of bar codes. Nature.

[ref-74] Lanfear R, Kokko H, Eyre-Walker A (2014). Population size and the rate of evolution. Trends in Ecology & Evolution.

[ref-75] Layton KKS, Martel AL, Hebert PDN (2014). Patterns of DNA barcode variation in Canadian marine molluscs. PLoS ONE.

[ref-76] Lebour MV (1935). The Breeding of *Littorina neritoides*. Journal of the Marine Biological Association of the United Kingdom.

[ref-77] Lee HJ, Boulding EG (2007). Mitochondrial DNA variation in space and time in the northeastern Pacific gastropod, *Littorina keenae*. Molecular Ecology.

[ref-78] Lee HJ, Boulding EG (2009). Spatial and temporal population genetic structure of four northeastern Pacific littorinid gastropods: the effect of mode of larval development on variation at one mitochondrial and two nuclear DNA markers. Molecular Ecology.

[ref-79] Leffler EM, Bullaughey K, Matute DR, Meyer WK, Ségurel L, Venkat A, Andolfatto P, Przeworski M (2012). Revisiting an old riddle: what determines genetic diversity levels within species?. PLoS Biology.

[ref-80] Lessios HA, Kane J, Robertson DR (2003). Phylogeography of the pantropical sea urchin *Tripneustes*: contrasting patterns of population structure between oceans. Evolution.

[ref-81] Li W-H (1977). Distribution of nucleotide differences between two randomly chosen cistrons in a finite population. Genetics.

[ref-82] Librado P, Rozas J (2009). DnaSP v5: a software for comprehensive analysis of DNA polymorphism data. Bioinformatics.

[ref-83] Lysaght AM (1941). The biology and trematode parasites of the gastropod *Littorina neritoides* (L.) on the Plymouth breakwater. Journal of the Marine Biological Association of the United Kingdom.

[ref-84] Mao Y, Gao T, Yanagimoto T, Xiao Y (2011). Molecular phylogeography of *Ruditapes philippinarum* in the Northwestern Pacific Ocean based on COI gene. Journal of Experimental Marine Biology and Ecology.

[ref-85] Marko PB, Barr KR (2007). Basin-scale patterns of mtDNA differentiation and gene flow in the bay scallop *Argopecten irradians concentricus*. Marine Ecology Progress Series.

[ref-86] Maruyama T, Nei M (1981). Genetic variability maintained by mutation and overdominant selection in finite populations. Genetics.

[ref-87] Mather KC (1955). Polymorphism as an outcome of disruptive selection. Evolution.

[ref-88] Miller MA, Pfeiffer W, Schwartz T (2010). Creating the CIPRES Science Gateway for inference of large phylogenetic trees. Gateway Computing Environments Workshop (GCE).

[ref-89] Mulligan CJ, Kitchen A, Miyamoto MM (2006). Comment on “Population size does not influence mitochondrial genetic diversity in animals”. Science.

[ref-90] Nabholz B, Glémin S, Galtier N (2009). The erratic mitochondrial clock: variations of mutation rate, not population size, affect mtDNA diversity across birds and mammals. BMC Evolutionary Biology.

[ref-91] Nabholz B, Mauffrey J-F, Bazin E, Galtier N, Glemin S (2008). Determination of mitochondrial genetic diversity in mammals. Genetics.

[ref-92] Navascués M, Emerson BC (2009). Elevated substitution rate estimates from ancient DNA: model violation and bias of Bayesian methods. Molecular Ecology.

[ref-93] Nei M (1987). Molecular evolutionary genetics.

[ref-94] Nei M, Miller JC (1990). A simple method for estimating average number of nucleotide substitutions within and between populations from restriction data. Genetics.

[ref-95] Nielsen R, Slatkin M (2013). An introduction to population genetics: theory and applications.

[ref-96] Nuryanto A, Kochzius M (2009). Highly restricted gene flow and deep evolutionary lineages in the giant clam *Tridacna maxima*. Coral Reefs.

[ref-97] Panova M, Blakeslee AMH, Miller AW, Mäkinen T, Ruiz GM, Johannesson K, André C (2011). Glacial history of the North Atlantic marine snail, *Littorina saxatilis*, inferred from distribution of mitochondrial DNA lineages. PLoS ONE.

[ref-98] Penny D (2005). Relativity for molecular clocks. Nature.

[ref-99] Piganeau G, Eyre-Walker A (2009). Evidence for variation in the effective population size of animal mitochondrial DNA. PLoS ONE.

[ref-100] Pons J, Barraclough TG, Gomez-Zurita J, Cardoso A, Duran DP, Hazell S, Kamoun S, Sumlin WD, Vogler AP (2006). Sequence-based species delimitation for the DNA taxonomy of undescribed insects. Systematic Biology.

[ref-101] Pons O, Petit RJ (1995). Estimation, variance and optimal sampling of gene diversity. I. Haploid locus. Theoretical and Applied Genetics.

[ref-102] Pons O, Petit RJ (1996). Measuring and testing genetic differentiation with ordered versus unordered alleles. Genetics.

[ref-103] Popovic I, Marko PB, Wares JP, Hart MW (2014). Selection and demographic history shape the molecular evolution of the gamete compatibility protein bindin in *Pisaster* sea stars. Ecology and Evolution.

[ref-104] Puillandre N, Lambert A, Brouillet S, Achaz G (2012). ABGD, Automatic Barcode Gap Discovery for primary species delimitation. Molecular Ecology.

[ref-105] Rambaut A, Suchard MA, Xie D, Drummond AJ (2014). http://beast.bio.ed.ac.uk/Tracer.

[ref-106] Ratnasingham S, Hebert PDN (2007). BOLD: the barcode of life data system (http://www.barcodinglife.org). Molecular Ecology Notes.

[ref-107] Reid DG, Dyal P, Williams ST (2010). Global diversification of mangrove fauna: a molecular phylogeny of *Littoraria* (Gastropoda: Littorinidae). Molecular Phylogenetics and Evolution.

[ref-108] Reid DG, Dyal P, Williams ST (2012). A global molecular phylogeny of 147 periwinkle species (Gastropoda, Littorininae). Zoologica Scripta.

[ref-109] Reid DG, Lal K, Mackenzie-Dodds J, Kaligis F, Littlewood DTJ, Williams ST (2006). Comparative phylogeography and species boundaries in *Echinolittorina* snails in the central Indo-West Pacific. Journal of Biogeography.

[ref-110] Reid DG, Rumbak E, Thomas RH (1996). DNA., morphology and fossils: phylogeny and evolutionary rates of the gastropod genus *Littorina*. Philosophical Transactions of the Royal Society of London B: Biological Sciences.

[ref-111] Robinson JD, Diaz-Ferguson E, Poelchau MF, Pennings S, Bishop TD, Wares J (2010). Multiscale diversity in the marshes of the Georgia coastal ecosystems LTER. Estuaries and Coasts.

[ref-112] Rogers AR (1995). Genetic evidence for a Pleistocene population explosion. Evolution.

[ref-113] Ronquist F, Teslenko M, Van der Mark P, Ayres DL, Darling A, Höhna S, Larget B, Liu L, Suchard MA, Huelsenbeck JP (2012). MrBayes 3.2: efficient Bayesian phylogenetic inference and model choice across a large model space. Systematic Biology.

[ref-114] Rueffler C, Van Dooren TJM, Leimar O, Abrams PA (2006). Disruptive selection and then what?. Trends in Ecology & Evolution.

[ref-115] Schneider S, Excoffier L (1999). Estimation of past demographic parameters from the distribution of pairwise differences when the mutation rates vary among sites: application to Human mitochondrial DNA. Genetics.

[ref-116] Seo T-K, Thorne JL, Hasegawa M, Kishino H (2002). Estimation of effective population size of HIV-1 within a host: a pseudomaximum-likelihood approach. Genetics.

[ref-117] Silva SE, Silva IC, Madeira C, Sallema R, Paulo OS, Paula J (2013). Genetic and morphological variation in two littorinid gastropods: evidence for recent population expansions along the East African coast. Biological Journal of the Linnean Society.

[ref-118] Simon C, Frati F, Beckenbach A, Crespi B, Liu H, Flook P (1994). Evolution, weighting, and phylogenetic utility of mitochondrial gene sequences and a compilation of conserved polymerase chain reaction primers. Annals of the Entomological Society of America.

[ref-119] Small KS, Brudno M, Hill MM, Sidow A (2007). Extreme genomic variation in a natural population. Proceedings of the National Academy of Sciences of the United States of America.

[ref-120] Smith BT, Klicka J (2013). Examining the role of effective population size on mitochondrial and multilocus divergence time discordance in a songbird. PLoS ONE.

[ref-121] Subramanian S, Lambert DM (2011). Time dependency of molecular evolutionary rates? Yes and no. Genome Biology and Evolution.

[ref-122] Tajima F (1989). Statistical method for testing the neutral mutation hypothesis by DNA polymorphism. Genetics.

[ref-123] Tamura K, Stecher G, Peterson D, Filipski A, Kumar S (2013). MEGA6: Molecular Evolutionary Genetics Analysis Version 6.0. Molecular Biology and Evolution.

[ref-124] Terranova MS, Lo Brutto S, Arculeo M, Mitton JB (2007). A mitochondrial phylogeography of *Brachidontes variabilis* (Bivalvia: Mytilidae) reveals three cryptic species. Journal of Zoological Systematics and Evolutionary Research.

[ref-125] Thomas JA, Welch JJ, Lanfear R, Bromham L (2010). A generation time effect on the rate of molecular evolution in invertebrates. Molecular Biology and Evolution.

[ref-126] Thompson JD, Higgins DG, Gibson TJ (1994). CLUSTAL W: improving the sensitivity of progressive multiple sequence alignment through sequence weighting, position-specific gap penalties and weight matrix choice. Nucleic Acids Research.

[ref-127] Turner TF, McPhee MV, Campbell P, Winemiller KO (2004). Phylogeography and intraspecific genetic variation of prochilodontid fishes endemic to rivers of northern South America. Journal of Fish Biology.

[ref-128] Uthicke S, Benzie JAH (2003). Gene flow and population history in high dispersal marine invertebrates: mitochondrial DNA analysis of *Holothuria nobilis* (Echinodermata: Holothuroidea) populations from the Indo-Pacific. Molecular Ecology.

[ref-129] Van den Broeck H, Breugelmans K, De Wolf H, Backeljau T (2008). Completely disjunct mitochondrial DNA haplotype distribution without a phylogeographic break in a planktonic developing gastropod. Marine Biology.

[ref-130] Vergara-Chen C, González-Wangüemert M, Marcos C, Pérez-Ruzafa Á (2010). Genetic diversity and connectivity remain high in *Holothuria polii* (Delle Chiaje 1823) across a coastal lagoon-open sea environmental gradient. Genetica.

[ref-131] Vollmer SV, Palumbi SR (2007). Restricted gene flow in the Caribbean staghorn coral *Acropora cervicornis*: implications for the recovery of endangered reefs. Journal of Heredity.

[ref-132] Wan Q-H, Wu H, Fujihara T, Fang S-G (2004). Which genetic marker for which conservation genetics issue?. Electrophoresis.

[ref-133] Wares JP, Cunningham CW (2001). Phylogeography and historical ecology of the North Atlantic intertidal. Evolution.

[ref-134] Wares JP, Goldwater DS, Kong BY, Cunningham CW (2002). Refuting a controversial case of a human-mediated marine species introduction. Ecology Letters.

[ref-135] Waters JM, McCulloch GA, Eason JA (2007). Marine biogeographical structure in two highly dispersive gastropods: implications for trans-Tasman dispersal. Journal of Biogeography.

[ref-136] Watterson G (1975). On the number of segregating sites in genetical models without recombination. Theoretical Population Biology.

[ref-137] Wecker P, Fournier A, Bosserelle P, Debitus C, Lecellier G, Berteaux-Lecellier V (2015). Dinoflagellate diversity among nudibranchs and sponges from French Polynesia: insights into associations and transfer. Comptes Rendus Biologies.

[ref-138] Williams ST, Reid DG (2004). Speciation and diversity on tropical rocky shores: a global phylogeny of snails of the genus *Echinolittorina*. Evolution.

[ref-139] Williams ST, Reid DG, Littlewood DTJ (2003). A molecular phylogeny of the Littorininae (Gastropoda: Littorinidae): unequal evolutionary rates, morphological parallelism, and biogeography of the Southern Ocean. Molecular Phylogenetics and Evolution.

[ref-140] Zeng K, Fu Y-X, Shi S, Wu C-I (2006). Statistical tests for detecting positive selection by utilizing high-frequency variants. Genetics.

[ref-141] Zhang J, Kapli P, Pavlidis P, Stamatakis A (2013). A general species delimitation method with applications to phylogenetic placements. Bioinformatics.

