# Supplementary material for: Mitochondrial DNA hyperdiversity and its potential causes in the marine periwinkle Melarhaphe neritoides (Mollusca: Gastropoda)
Source: PeerJ. 2016 Oct 5;4:e2549. doi: 10.7717/peerj.2549 (PMC5068447; doi:10.7717/peerj.2549)
Supplement: Table S2 — N, number of individuals; N1, N2, N3, N4, number of individuals used in dataset 1, dataset 2, dataset 3 and dataset 4 respectively. [file peerj-04-2549-s002.docx]

*The following supplement accompanies the article*

**Mitochondrial DNA hyperdiversity and its potential causes in the marine periwinkle *Melarhaphe neritoides* (Mollusca: Gastropoda)**

Séverine Fourdrilis^*^, Patrick Mardulyn, Olivier J. Hardy, Kurt Jordaens, Antonio M. de Frias Martins Thierry Backeljau

* Corresponding author: sfourdrilis@naturalsciences.be

Table S2. Specimens samples and datasets used in this study.

| Locality | *N* | sampling date | *N1* | *N2* | *N3* | *N4* |
| --- | --- | --- | --- | --- | --- | --- |
| FAI | 43 | 06/28/1993 | 42 |  | 43 | 43 |
| FAI | 46 | 07/06/2012 |  |  | 46 |  |
| FLO | 42 | 1992 | 39 |  | 42 | 42 |
| FLO | 45 | 07/10/2012 |  |  | 45 |  |
| MOS | 223 | 06/29/2012 |  | 223 |  |  |
| PIC | 45 | 10/14/1993 | 37 |  | 45 | 45 |
| PIC | 43 | 07/04/2012 |  |  | 43 |  |
| SMA | 43 | 04/17/1996 | 32 |  |  | 43 |
| SMI | 39 | 07/31/1993 | 35 |  | 39 | 39 |
| SMI | 41 | 06/28/2012 |  |  | 41 |  |
| **Total** | 610 |  | 185 | 223 | 344 | 212 |

*N*, number of individuals; *N1*, *N2*, *N3*, *N4*, number of individuals used in dataset 1, dataset 2, dataset 3 and dataset 4 respectively.
