# Supplementary material for: Mitochondrial DNA hyperdiversity and its potential causes in the marine periwinkle Melarhaphe neritoides (Mollusca: Gastropoda)
Source: PeerJ. 2016 Oct 5;4:e2549. doi: 10.7717/peerj.2549 (PMC5068447; doi:10.7717/peerj.2549)
Supplement: Table S3 — n, number of sequences used; d1, number of base differences per site (p-distance) from averaging over all sequence pairs within each group ± standard error; n/c, cases in which it was not possible to estimate evolutionary distances; d2, number of base differences per site (p-distance) from averaging over all sequence pairs between groups (under diagonal) and standard error estimates (above diagonal). [file peerj-04-2549-s003.docx]

*The following supplement accompanies the article*

**Mitochondrial DNA hyperdiversity and its potential causes in the marine periwinkle *Melarhaphe neritoides* (Mollusca: Gastropoda)**

Séverine Fourdrilis^*^, Patrick Mardulyn, Olivier J. Hardy, Kurt Jordaens, Antonio M. de Frias Martins Thierry Backeljau

* Corresponding author: sfourdrilis@naturalsciences.be

Table S3. Estimates of average evolutionary divergence over COI sequence pairs within and between groups.

| Species | n | d_1_ | d_2_ | | | | | | | | | |
| --- | --- | --- | --- | --- | --- | --- | --- | --- | --- | --- | --- | --- |
|  |  |  | | [1] | [2] | [3] | [4] | [5] | [6] | [7] | [8] | [9] |
| [1] *Bembicium auratum* | 1 | n/c | |  | 0.017 | 0.015 | 0.016 | 0.018 | 0.017 | 0.018 | 0.016 | 0.017 |
| [2] *Cremnoconchus syhadrensis* | 1 | n/c | | 0.196 |  | 0.017 | 0.017 | 0.018 | 0.017 | 0.018 | 0.016 | 0.018 |
| [3] *Lacuna pallidula* | 2 | 0.000 ± 0.000 | | 0.166 | 0.212 |  | 0.016 | 0.017 | 0.017 | 0.017 | 0.016 | 0.017 |
| [4] *Laevilitorina caliginosa* | 1 | n/c | | 0.166 | 0.190 | 0.174 |  | 0.018 | 0.017 | 0.018 | 0.016 | 0.017 |
| [5] *Littorina littorea* | 19 | 0.004 ± 0.001 | | 0.224 | 0.228 | 0.212 | 0.219 |  | 0.017 | 0.016 | 0.017 | 0.019 |
| [7] *Peasiella isseli* | 1 | n/c | | 0.212 | 0.230 | 0.200 | 0.198 | 0.214 |  | 0.018 | 0.017 | 0.018 |
| [9] *Tectarius striatus* | 53 | 0.006 ± 0.001 | | 0.202 | 0.246 | 0.185 | 0.222 | 0.187 | 0.225 |  | 0.017 | 0.019 |
| [6] *Melarhaphe neritoides* | 213 | 0.018 ± 0.002 | | 0.181 | 0.201 | 0.172 | 0.176 | 0.223 | 0.204 | 0.212 |  | 0.017 |
| [8] *Pomatias elegans* | 8 | 0.009 ± 0.002 | | 0.209 | 0.244 | 0.199 | 0.203 | 0.254 | 0.231 | 0.271 | 0.228 |  |

n, number of sequences used; d_1_, number of base differences per site (p-distance) from averaging over all sequence pairs within each group ± standard error; n/c, cases in which it was not possible to estimate evolutionary distances; d_2_, number of base differences per site (p-distance) from averaging over all sequence pairs between groups (under diagonal) and standard error estimates (above diagonal).
